# Participants’ perspectives and preferences on clinical trial result dissemination: The TRUST Thyroid Trial experience

**DOI:** 10.12688/hrbopenres.12817.2

**Published:** 2019-03-22

**Authors:** Emmy Racine, Caroline Hurley, Aoife Cheung, Carol Sinnott, Karen Matvienko-Sikar, Christine Baumgartner, Nicolas Rodondi, William H. Smithson, Patricia M. Kearney

**Affiliations:** 1 School of Public Health, University College Cork, Cork, Ireland; 2 The Healthcare Improvement Studies (THIS) Institute, University of Cambridge, Cambridge, UK; 3 Department of General Internal Medicine, Inselspital, Bern University Hospital, University of Bern, Bern, Switzerland; 4 Institute of Primary Health Care (BIHAM), University of Bern, Bern, Switzerland; 5 Department of General Practice, University College Cork, Cork, Ireland

**Keywords:** Patient and public involvement, patient involvement in clinical trials, study within a trial, SWAT, Clinical trial result dissemination, study results, research dissemination, trial results.

## Abstract

**Background**: While there is an increasing consensus that clinical trial results should be shared with trial participants, there is a lack of evidence on the most appropriate methods. The aim of this Study Within A Trial (SWAT) is to use a patient and public involvement (PPI) approach to identify, develop and evaluate a patient-based approach to receiving trial results for participants in the Thyroid Hormone Replacement for Subclinical Hypo-Thyroidism Trial (TRUST), a trial of thyroxine versus placebo in people aged 65 years and older.

**Methods**: Mixed methods study with three consecutive phases. Phase 1 iteratively developed a patient-based approach using semi-structured focus groups and a consensus-orientated-decision model, a PPI group to refine the method and adult literacy review for plain English assessment. Phase 2 was a single-blind parallel group trial. Irish TRUST participants were randomised to the intervention (patient-based approach) and control group (standard approach developed by lead study site). Phase 3 used a patient understanding questionnaire to compare patient understanding of results between the two groups.

**Results**: Participants want to receive results of clinical trials, with qualitative findings indicating three key themes including ‘acknowledgement of individual contribution’, ‘contributing for a collective benefit’ and ‘receiving accessible and easy to understand results’. Building on these findings, the patient-based approachwas developed. TRUST participants (n=101) were randomised to the intervention (n=51) or control group (n=50). The questionnaire response rate was 74% for the intervention group and 62% for the control group.  There were no differences in patient understanding between the two approaches.

**Conclusions**: We have demonstrated that it is feasible to involve trial participants in the development of result dissemination materials. Although, in this study PPI did not influence patients’ understanding of results, it documents the process of conducting PPI within the clinical trial setting.

## Introduction

Patient and public involvement (PPI) is increasingly recognised as an essential component of clinical research. In the UK, the national advisory group supporting active public involvement in health services, public health and social care research (INVOLVE) defines PPI as ‘research being carried out ‘with’ or ‘by’ members of the public rather than ‘to’ ‘about’ or ‘for’ them’ ^1^. In clinical trials, PPI has been defined as experimenting with participants instead of experimenting on participants ^2^. PPI may occur at any stage during the research process from priority setting and drafting study protocols right through to conducting the study, interpreting the end results and communicating and disseminating research findings ^3, 4^. Research funders increasingly expect that PPI is prioritised and resourced within studies. This increasing expectation has heightened the risk of researchers carrying out ‘tick-box’ PPI rather than ‘meaningful’ involvement ^5^. There are many moral and ethical arguments being made for PPI. Many believe that as citizens and taxpayers, members of the public have a right to influence research that is being funded by public money ^6^. PPI researchers are also making pragmatic arguments for PPI and providing anecdotal accounts about how PPI can make research more relevant, accessible and acceptable to participants ^7^. The ethical arguments are often seen as sufficient regardless of any pragmatic impact. However, PPI costs time and money, therefore pragmatic claims need scrutiny ^8^. More substantive evidence is needed to evaluate the potential impact of PPI on the conduct and outcomes of research ^5, 9^. In 2001, the need to establish if PPI leads to actual, rather than merely perceived benefits for research processes and output was identified. Over fifteen years later, this need remains.

In clinical research, the results of clinical trials have not traditionally been shared with clinical trial participants. A recent survey carried out on a large registry of health research participants, found that while 95.6% of respondents said researchers should always or sometimes offer the results to participants, only 33% of respondents actually received the results of studies in which they had participated ^10^. An upcoming European Union Clinical Trial Regulation requires sponsors to provide summary results of clinical trials in a format understandable to laypersons, including participants ^11^. However, there is a lack of evidence on the most appropriate methods of sharing results with participants. Uncertainty persists around what information should be shared, how results should be shared and who should be responsible for sharing the results. Since the findings of clinical research often exist in a complex context of scientific exchange and debate, it is important that the information shared is accessible and relevant to participants ^12^. The increasing understanding of the importance of sharing research results with study participants is somewhat linked to a wider movement towards transparency in trials. This movement is largely promoted by initiatives such as SPIRIT, CONSORT and AllTrials. The SPIRIT Statement provides guidance to researchers to improve the completeness and quality of trial protocols ^13^, the Consolodated Standards of Reporting Trials (CONSORT) Statement is an evidence based, minimum set of recommendations for reporting randomised trials ^14^ and the AllTRials iniative calls for all past and present triasl to be registered and their full methods and summary results reported ^15^. Some of these initiatives also include recommendations for disseminating results to research participants. For example, the SPIRIT statement states that study results must be released to participating physicians, referring physicians, patients and the general medical community ^13^.

The Thyroid Hormone Replacement for Subclinical Hypothyroidism Trial (TRUST) was a multi-centre, double blind, placebo controlled, phase III clinical trial testing the efficacy of thyroxine replacement in subclinical hypothyroidism in older community dwelling adults ^16^. The results of the TRUST trial were published in the New England Journal of Medicine on 3 ^rd^ of April, 2017 ^16^. This Study Within A Trial (SWAT) was conducted at the Irish TRUST trial site prior to and after publication of results. The aim of this SWAT was to investigate methods of disseminating trial findings to participants by using a PPI approach to identify, develop and evaluate a patient-based approach of receiving trial results.

## Methods

### Study design

This was a sequential mixed methods study with three phases. In this study, methods were combined for complementarity, where each method addressed a different aspect of the study aim ^17^. The first phase used a qualitative approach to identify and develop a patient-based approach to disseminating the results, the second phase used a SWAT intervention to compare the dissemination approaches and the third phase used a quantitative patient understanding questionnaire to evaluate the patient-based approach. The full study protocol has been published elsewhere ^18^, but a summary follows here.

### Setting

The study sites for the TRUST trial were the University of Glasgow, Scotland (lead site); Leiden Academy on Vitality and Ageing, The Netherlands; Leiden University Medical Centre, The Netherlands; University of Berne, Switzerland; and University College Cork, Ireland. A total of 738 participants with subclinical hypothyroidism were recruited to the trial over a three-and-a-half year period from 2013–2017 ^16^. The trial completed recruitment in November 2016 and the results were published in April 2017 ^16^.

This SWAT was conducted at the Irish TRUST site. The hub centre for the Irish TRUST site was located at the Mercy University Hospital, Cork where 38 participants were recruited. A further 77 participants were recruited from five satellite sites.

### Population

As this SWAT was embedded in an ongoing clinical trial the study sample was determined by the TRUST Thyroid trial. There were 115 TRUST participants recruited in the Irish site, 11 of these participants withdrew over the course of the trial. Our study sample included all remaining TRUST participants (n=104).

## Phase One: Identification and development of patient-based approach (qualitative and PPI phase)

The first phase of the study used a qualitative approach to iteratively identify and develop a patient-based approach to disseminate the results of TRUST trial. This was done in three separate stages: qualitative focus groups, a PPI group and an adult literacy review.

### Focus groups

Three semi-structured focus groups were conducted with four to eight TRUST trial participants per group. All Cork-based patients (n = 38) were contacted via letter and invited to participate. A €20 shopping voucher was given to all participants to cover travel expenses. Each session was led by trained qualitative researchers (WHS, ER, CH). A topic guide was used to guide the focus groups. The topic guide was reviewed and refined by all members of the SWAT research team (see Supplementary File 1: Focus group topic guide).

The Consensus-Oriented-Decision-Making (CODM) model was used to guide the group to reach a consensus ^19^. The CODM model is accepted as a flexible model for reaching decisions ^19^. In this study some of the steps were initiated by the focus group facilitator and others occurred naturally as a follow on from the previous step. Below is an outline of each of the seven steps of the CODM model and how they were used in this study:

1. Framing the topic: The focus group facilitator introduced the idea of sharing results with participants and provided some context on the reasons why results are/ are not shared with participants.
2. Open discussion: The facilitator asked the group whether or not they think results should be shared with trial participants and whether or not they would like to receive the results of the TRUST trial.
3. Identifying underlying concerns: The previous discussion naturally followed on to participants asking questions and expressing concerns about the result method, content and language that would be used.
4. Collaborative proposal building: The group worked together to agree on the important elements of the results in terms of result method, content and language.
5. Choosing a direction: This step occurred naturally as part of the previous step.
6. Synthesizing a final proposal: The facilitator re-iterated the proposal the group had agreed upon and asked the group for feedback.
7. Closure: This step occurred naturally as part of the previous step.

***Analysis.*** Focus group recordings were transcribed verbatim and entered into NVivo Version 11 for data management during thematic analysis. Braun and Clarke guidelines ^20^ for conducting thematic analysis were followed. Initial focus group transcripts were analysed independently by two researchers (ER and AC). Each transcript was read multiple times (data familiarisation) and initial codes were identified. These codes were then used to identify emerging themes. Both researchers discussed emerging themes and conducted further refinement. The refined themes were then discussed and agreed upon with other members of the research team (ER, CH, AC, KMS). Researchers (ER, CH, AC) then used the focus group findings to develop an initial draft of a patient-based approach for the dissemination of results (see Supplementary File 2: Draft one of patient-based result letter).

### PPI group

A PPI group was established to develop and refine the content of the patient-based appproach for the dissemination of results. During the focus groups, three TRUST trial participants volunteered to take part in the PPI group. In addition to these three PPI partners, an additional partner was identified from a previous qualitative research study undertaken by the research team. This individual was keen to learn more about research and expressed an interest in being involved in future projects. While this individual had previous experience of taking part in research (as an interview participant), she had no experience of taking part in a clinical trial or being involved as a PPI partner. Originally, we intended to conduct these sessions in a group format, due to difficulties with PPI partners’ schedule commitments, one-to-one sessions were conducted. At the one-one session, a researcher (ER) and the PPI partner discussed the layout, content and language of the initial draft of the result method. Researchers and PPI partners worked together to edit different sections of the document. These discussions were not audio recorded but comprehensive field notes were taken by the researcher (ER). These notes were then collated by the researcher and used to further ensure that the results letter reflected PPI partners’ perspectives and preferences.

### Adult literacy review

While the PPI group had significant input into the format and language used in the patient-based approach, the research team felt that it would be of additional benefit to collaborate with the National Adult Literacy Agency (NALA) to ensure the document adhered to national “Plain English” standards. These standards ensured that the information presented to trial participants was sufficiently easy to read and understand (literacy). This would help to ensure that trial participants were able to make sound health decisions based on the information presented (health literacy) ^21^. This review was an iterative process with several drafts exchanged for editing. Although the review was taken as an additional step to the published protocol for the study, the research team felt it was helpful to further ensure that the document was accessible and easy to understand.

At the end of the first phase of the study, a final draft of the patient-based result letter was approved by researchers, PPI group and adult literacy experts (see Supplementary File 3: Final draft of patient-based result letter).

## Phase Two: Dissemination of trial results (intervention phase)

The second phase of the study used a SWAT intervention to disseminate the results of the TRUST Thyroid Trial to trial participants. This was done using a prospective, randomised, single blind, parallel trial design. It is important to note that when the term randomisation is used, it refers to the allocation of patients to intervention/control within the SWAT and not the TRUST Thyroid trial. Irish TRUST participants were randomised to intervention or control groups using an online random number generator. The intervention group received the patient-based letter format (see Supplementary File 3: Final version patient-based results letter) and the control group received a copy of the TRUST results press release, which was made available by the lead study site on the TRUST Thyroid Trial Website (see Supplementary File 4: Standard results letter). Participants were blinded to their intervention group. One member of the research team was un-blinded in order to perform the randomisation and distribute the results of the trial. As they were un-blinded to perform these two important tasks, they were not involved in the data analysis or interpretation in any way.

## Phase Three: Evaluation of patient –based approach (quantitative phase)

The third phase of the study used a quantitative patient understanding questionnaire to evaluate the patient-based approach to disseminating trial results. The questionnaire was developed in consultation with experts in the area of subclinical hypothyroidism and scale (questionnaire) development (PK and KMS). The early development of the questionnaire was guided by a consultation document, which accompanies the EU Clinical Trials Regulation No 536/2014 ^22^. This document highlights the information which should be presented to trial participants in the trial summary at the end of a trial. However, initial questionnaire items were modified to allow for psychometric testing. The final questionnaire contained 12 questions; six items were measured on a five point LIKERT scale, there were four multiple-choice questions and two vignettes. The first six items measured patients’ perceived understanding of results, the four multiple choice measured patients’ actual understanding of results by requiring them to select the correct answer. To further test participants’ understanding of the trial results, two vignettes describing two typical patient case studies of older adults with subclinical hypothyroidism were provided with a question on whether a doctor should prescribe thyroxine for the hypothetical patient described. The questionnaire was reviewed by the PPI group to assess content and face validity. It then underwent further review by NALA to ensure adherence to the national ‘Plain English’ standard. The final version of the questionnaire can be seen in Supplementary File 5: Patient understanding questionnaire.

The questionnaire was sent to all Irish TRUST participants (intervention and control group) one week after they received the results of the trial. A reminder questionnaire was sent to non-responders 3 weeks later.

***Analysis.*** The primary outcome was the difference in levels of patient understanding between the intervention and control groups. This measured the impact of PPI on patient understanding of end of trial results. The psychometric properties and construct validity of the questionnaire were examined with exploratory factor analysis. Principal component analysis (PCA) was conducted on the six LIKERT scale items. Internal consistency of the questionnaire was investigated using Cronbach's alpha coefficient. Completed questionnaires were entered into SPSS software (version 24) and analysed using descriptive and inferential (Chi-square test and Fishers Exact) statistics. The researcher carrying out data input and analysis was blinded to the participants' allocation status.

### Costs of conducting PPI

The lead researcher (ER) kept a detailed account of all direct costs associated with conducting PPI for the purpose of this study. These costs included researcher salary, travel and expenses for PPI participants, adult literacy review and printing and postage costs.

This paper has been written in adherence to the Guidance for Reporting Involvement of Patients and Public 2 (GRIPP 2) ^23^. The GRIPP 2 checklist is a tool, developed to improve the reporting of patient and public involvement in research and guide the development of a transparent, consistent and high-quality PPI evidence base. The Good Reporting of a Mixed Methods Study (GRAMMS) framework was also used to inform the reporting of the findings ^24^.

## Results

Characteristics of the trial participants stratified by participation in the different stages of the study are presented in Table 1.

**Table 1.** Characteristics of trial participants stratified by participation in the different stages of the study.

	Total Irish TRUST participants (n=104)	Attended SWAT focus groups ^1^ (n=19) Total Sample n=38 RR ^2^ =50%	Randomised ^3^ (n=101)		Returned SWAT questionnaire (n=69) Total Sample n=101 RR ^2^=68%	
			Intervention Group (n=51)	Control Group (n=50)	Intervention Group (n=38) RR= 74%	Control Group (n=31) RR=62%
**Sex**						
Male	61 (58.7%)	14 (73.7%)	31 (60.8%)	28 (56%)	26 (68%)	16 (52%)
Female	43 (41.3)	5 (26.3%)	20 (39.2%)	22 (44%)	12 (32%)	15 (48%)
**Age**						
65–74	57 (54.8%)	12 (63.1%)	32 (62.7%)	24 (48%)	25 (66%)	12 (45%)
75+	47 (45.2%)	7 (36.9%)	19 (37.3%)	26 (52%)	13 (34%)	17 (55%)
**Education**						
Primary only	22 (21.2%)	2 (10.5%)	12 (23.6%)	9 (18%)	10 (26%)	8 (26%)
Secondary/Tertiary	47 (45.1%)	12 (63.2%)	24 (47.1%)	22 (44%)	19 (50%)	11 (35%)
Unknown	35 (33.7%)	5 (26.3%)	15 (29.3%)	19 (38%)	9 (24%)	12 (39%)

^1^A subgroup of Irish TRUST participants (n=38) were invited to focus groups.

^2^RR=Response Rate

^3^Total Irish TRUST participants (n=104) excluding PPI partners (n=3)= n=101.

## Phase One: Identification and development of patient-based approach (qualitative and PPI phase)

### Focus groups

Three focus groups were held with 19 out of 38 participants accepting an invitation to join. Participants who attended the focus groups were similar in age, gender, education level to those who did not attend.

Focus group findings indicate that participants want to receive the results of the trial in which they are taking part. Three main themes emerged in relation to participants’ perspectives of and preferences for receiving trial results: ‘acknowledgement of individual contribution’, ‘contributing for a collective benefit’ and ‘receiving accessible and easy to understand results’.

### Acknowledgement of individual contribution

Many participants reported feeling they had made an individual contribution to the trial in terms of their time and personal information while attending the trial study visits. As such, participants felt that receiving the results of the trial would provide an acknowledgement of this individual contribution:

*‘Yes, I mean it’s kind of instinctive… when you go into a [clinical trial] and you spend and invest that time in it. I mean okay I had the time to invest but you know at the end of the day, [receiving the result] is kind of like your pay off. ’* (FG2 P3)

### Contributing for a collective benefit

While participants spoke about making an individual contribution to the trial, they felt that their involvement contributed to a collective benefit or greater good. Participants reported that receiving the results of the trial would help them to feel that they had contributed to this greater good:

*‘I’m not really interested in my own personal results but as the results of the scheme as a whole. You know the idea is, does the study help or hinder old people and that’s what I want to know’* (FG2 P1)

This feeling of contributing for a collective benefit was further reinforced when participants discussed their desire to understand how the results of the trial will be implemented by medical experts and ultimately how it will affect others who have the condition:

*‘I would like to know, if they found out, okay, do we treat these people or not. That would be good. Do we treat them or don’t we treat them? I think that is what it’s all about’ (FG3 P4)*

### Receiving accessible and easy to understand results

Participants expressed a clear need to receive the results of the trial in an accessible and easy to understand way. This preference applied to the format, language and content of the patient-based approach.

The majority of participants said they would like to receive the results in a letter format posted to them directly from the TRUST trial. Participants felt that this method would be accessible to them as they could read the results *‘in text’* (FG3 P4) and keep a *‘hardcopy’* (FG P1). While participants wanted an official statement of the results in a letter format, they also felt it was important to add a personal element to the letter. They suggested this could be done by offering participants a phone number that they could call if they wished to discuss any further issues or concerns with the TRUST study team:

*‘Could you attach a helpline on to it? If you know, somebody had some kind of serious medical question or that they thought was a bit personal element or whatever. That they’d like to talk to a medical person or whatever. Instead of just talking to your GP, maybe that would add another dimension of care around the TRUST’* (FG2 P3)

Participants agreed that the format, content and language of the results letter needed to be easy to read and understand. All participants wanted the letter to be no longer than 2–3 pages and presented in a question and answer format. Participants believed the content of the results letter should include *‘pertinent information’* (FG1 P7) relating to the trial itself, the study drug (including side effects) and the results of the trial. They stressed the importance that this information needed to be informed by medical experts and *‘from a good authoritative source’* (FG2 P2) but it should be presented to them in a language that fits their current context and could be easily understood by those who do not have scientific or medical backgrounds.

*‘Just in ordinary language that we can understand ourselves, you know that we don’t want big and long explanation or that, just that we can pick it up straight away that it’s without any huge number of pages. Just the bare, to me anyway, answers to the questions.’* (FG3- P2)

It was evident from the focus groups that participants want to receive the results of the trial both to acknowledge their individual contribution to the trial and also help them to feel that they had contributed to a greater good. Participants expressed a clear preference to receive the results in an accessible and easy to understand way. These results were used by the researcher (ER) to develop an initial draft of the results letter (see Supplementary File 2: Draft one patient-based result letter).

### PPI group

The initial draft of the results letter was then further iteratively developed by the PPI group. There were four PPI partners in total (three trial participants and one older adult) Each partner toook part in one-to-one session. Each session contained an open discussion between the researcher (ER) and PPI partners on the layout, content and language of the document. Researchers and PPI partners worked together to write, re-write, edit and change different sections of the document.

### Health literacy review

This draft was then iteratively reviewed and approved by health literacy experts from the NALA (see Supplementary File 3: Final version patient-based results letter).

## Phase Two: Dissemination of trial results (intervention phase)

There were a total of 101 Irish TRUST participants randomised to the SWAT intervention. Trial participants from the PPI group (n = 3) were excluded from randomisation as they reviewed the content of the intervention method prior to the intervention. The intervention group (n=51) received the patient-based letter format (see Supplementary File 3: Final version patient-based results letter) and the control group (n=50) received a copy of the TRUST results press release, which was made available by the lead study site on the TRUST Thyroid Trial Website (see Supplementary File 4: Standard results letter).

## Phase Three: Evaluation of patient-based approach (quantitative phase)

The overall response rate for the patient understanding questionnaire was 68% (69/101). The response rate for the intervention group was 74% (38/51) and the response rate for the control group was 62% (31/50). There were no significant differences in age, gender and education between those who returned the questionnaire and those who did not.

Post hoc power calculations showed that the study was underpowered to detect an effect. Power for each of the patient understanding components ranged from .01 to. 58.

Table 2 below shows the results of patients’ perceived understanding of the purpose and context of the TRUST Thyroid Trial. Due to low participant numbers across the five Likert responses, the questionnaire response bands have been contracted from ‘Strongly Agree’ and ‘Agree’ to ‘Yes, ‘Strongly Disagree’ and ‘Disagree’ to ‘No’ and ‘Neither agree nor disagree’ to ‘Neutral’. The results show that patients’ perceptions of understanding are similar between the intervention and control groups. Subgroup analysis showed patient’s understanding was not significantly impacted by age, gender or educational level.

**Table 2.** Patient perceptions of understanding presented by group ^1^.

Item	Group	Yes	No	Neutral	p-value
I understand why the TRUST Thyroid Trial took place.	Intervention (n=38)	37 (97.4%)	1 (2.6%)	0 (0%)	0.584
	Control (n=31)	29 (93.5%)	2 (6.5%)	0 (0%)	
I understand why I was invited to the TRUST Thyroid Trial	Intervention (n=38)	38 (100%)	0 (0%)	0 (0%)	0.198
	Control (n=31)	29 (93.5%)	2 (6.5%)	0 (0%)	
I know why the medicine Levothyroxine is used to treat subclinical hypothyroidism	Intervention (n=38)	32 (84.2%)	2 (5.3%)	4 (10.5%)	0.893
	Control (n=31)	25 (80.6%)	3 (9.7%)	3 (9.7%)	
I am aware of the side effects of Levothyroxine	Intervention (n=38)	30 (78.9%)	5 (13.2%)	3 (7.9%)	0.090
	Control (n=31)	17 (54.8%)	7 (22.6%)	7 (22.6%)	
I understand the impact of Levothyroxine on thyroid specific quality of life	Intervention (n=38)	31 (81.6%)	5 (13.2%)	2 (5.3%)	0.281
	Control (n=31)	20 (64.5%)	7 (22.6%)	4 (12.9%)	
I understand how doctors will use the results of the TRUST Thyroid trial to treat people with subclinical hypothyroidism	Intervention (n=38)	33 (86.8%)	2 (5.3%)	3 (7.9%)	0.878
	Control (n=31)	26 (83.9%)	3 (9.7%)	2 (6.5%)	

^1^Patient perceptions of understanding were assessed using a five point LIKERT scale.

**Figure 1.**
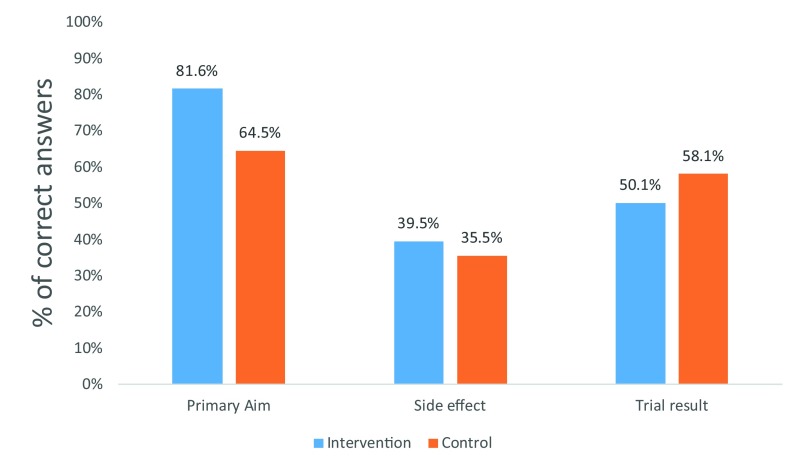
Patient understanding of primary aim, side effect and trial result of the TRUST Thyroid Trial presented by group ^1^. ^1^Patient understanding of primary aim, side effect and trial result was assessed using multiple choice questions.

Figure 1 shows patients’ actual understanding of the primary aim, side effect and results of the TRUST Thyroid Trial. Almost 82% (n=31) of the intervention group and 65% (n=20) of the control group correctly understood the primary aim of the TRUST trial (p=0.108). Almost 40% (n=15) of the intervention group and 36% (n=9) of the control group correctly understood the associated side effects of the active drug (p=0.734). In total 50% of the intervention group (n=19) and 58% of the control group correctly understood the results of the trial (p=0.504). There were no differences in patient understanding of trial results between the intervention and control groups.

In terms of patient understanding of hypothetical patient case studies, 43% (n=13) of the intervention group gave the correct answer to Vignette A; this was lower than the control group (62.1%, n=18, p=0.15). In total 77% (n=23) of the intervention group gave the correct answer to Vignette B, this was higher than the control group (66%, n=19, p=0.344).

### Psychometric testing

An exploratory principal components analysis (PCA) was conducted on the patient understanding questionnaire to determine its usefulness as a measure of perceived understanding. The Kaiser-Meyer-Olkin (KMO) measure verified the sampling adequacy for the analysis, KMO= .83. Bartlett’s test of sphericity indicated that the correlation matrix was significantly different from an identity matrix, X2 (.852) = 283.92, p<.001. An examination of eigenvalues greater than Kaiser’s criterion of one, suggested the extraction of one factor; this was supported by inspection of Cattell’s scree plot. An examination of the constituent items for this factor structure also indicated that items loaded most highly on a single factor. This single factor represents a measure of perceived understanding of trial results. PCA was then conducted using an oblique (direct oblimin) rotation, specifying the extraction of one factor. This model explained a combined 69.58% of the variance in patients understanding of the TRUST thyroid trial.

### Cost of conducting PPI

The total cost of this study amounted to €8,049 (see Supplementary File 6: Costs of conducting PPI).

## Discussion

While PPI is increasingly recognised as an important element of clinical research, evidence on optimal methods and potential impact is lacking ^4, 9^. Previous research conducted on the impact of PPI has largely focused on the experiences of participants and researchers ^25^ and on the research process in broad terms ^26^. In this study, our primary outcome was specific: a quantitative measure of patient understanding of trial results between those who received the patient-based approach and the standard approach. To our knowledge there has been no previous research conducted on the impact of PPI on patient understanding of trial results.

The involvement of clinical trial participants in this study offered insightful perspectives on the information needs of the study population in terms of receiving end of trial results. Study findings show that trial participants want to receive the results of the clinical trial in which they had participated. This is supported by much of the available literature on patients’ preferences of receiving results, with up to 90% of participants in previous studies reporting a desire to receive results ^27^. Focus group findings showed that participants felt that receiving results would provide an acknowledgement of their individual contribution to the trial. This finding complements previous commentaries about result sharing being an ‘ethical imperative or ‘moral obligation’. Fernandez et al. points out that many participants place their trust in science and researchers owe a debt to participants to fulfil their trust and recognise their altruism ^12, 28^.

Unsurprisingly, findings also show that participants want to receive results that are accessible and easy to understand. In this study, the preferred format of receiving results was a letter posted to them directly from the TRUST trial. This preference is also consistent with the literature on patient preferences of receiving results. A previous study investigating prefrences of individuals taking part in a cardiac rehabilitation trial found that 80% of trial participants preferred to receive the results by post ^29^. The patient-based approach identified in this study was feasible for researchers to develop with significant involvement from trial participants and adult literacy experts.

Previous studies exploring participants’ reactions found that sharing trial results with participants can cause some negative impacts such as anxiety, anger, guilt, upset and confusion ^30– 32^. As far as researchers in this study are aware, providing results did not cause any negative impacts. This may have been due to the fact that the TRUST trial had a low risk of morbidity or mortality compared to some of the other studies citing negative impacts. Both result methods contained the telephone number, email address and postal address of the research team and participants were urged to contact should they have any questions or concerns relating to the study. The research team did not receive any queries.

Previous systematic reviews highlight the lack of evidence on economic analysis of PPI and call for researchers to consider the costs of its implementation ^26, 33^. As discussed previously research funders are increasingly demanding that PPI be carried out in research. However, the costs of PPI are often underestimated and can cause a significant financial burden on research project budgets ^26, 33– 35^. It is extremely important that researchers plan PPI at the grant proposal stage and estimate the costs appropriately. If these costs are not correctly estimated during the initial stages of developing research proposals, they may cause a financial burden on PPI partners.

Participants in this study were not paid for their time but were provided with a €20 voucher to cover travel expenses. When PPI is not the primary focus of a study, researchers do not consider the cost implications at the beginning of the study and are often tied with limited resources to carry out PPI ^34– 36^. INVOLVE, the national advisory group supporting active public involvement in health services, public health and social care research in the UK, have recommended that PPI partners should be paid for their involvement ^37^. Despite this, existing research suggests that institutional difficulties make negotiating the mechanisms of paying participants very difficult ^34^. One study reported that in order for participants to be remunerated for their efforts, they needed to be registered as employees, a process that incurred much paperwork and time delays ^34^. This study outlines the cost of conducting PPI and includes a full breakdown of costs (see Supplementary File 6: Costs of conducting PPI). This breakdown provides a template to other researchers who plan to carry out and evaluate PPI as part of their research. It is important to note that not all costs associated with carrying out the study were included in this amount. For example, the only salary costed was that of the research assistant. The expertise provided by other members of the study team were not included in the total cost as they were being paid by the University or other research grants. The total cost of conducting this study was €8,049 which is not insignificant but should be considered in the context of the cost of large-scale trials.

### Strengths and Limitations of the study

While this study provides important insights into patients’ preferences of receiving trial results, it is not without limitations. Firstly, existing PPI literature states that ‘to understand the research needs and challenges, PPI has to engage people who are able to offer perspectives from the study population’ ^3^. All PPI partners in this study were active members of the research community as they had taken part in the TRUST trial and had agreed to long-term follow up. This is a strength of the SWAT as they were able to offer perspectives from the study population, however it does have an important implication for their reporting of understanding the results of the trial. They may be more inclined to rate their understanding as high because of their investment in the trial ^38^, thus potentially minimising differences between the intervention and control conditions and minimising inferences that can be drawn about the intervention. Previous research suggests that people that actively choose to engage in research either as research participants or involvement partners are more likely to be middle-class and highly educated ^39, 40^. In this study, those that attended the focus groups and PPI group were similar in education level to those that did not attend. This is not surprising considering the entire study sample had already actively volunteered to take part in the TRUST trial.

Secondly, the results of the patient understanding questionnaire show that the levels of patient understanding were similar between the two groups. However, this study was underpowered to detect an effect. As this was a Study Within A Trial (SWAT), the power was limited by the sample size that was available to us from the trial (n=115). Furthermore, validation of the patient understanding questionnaire was limited by the sample size in this study. While validation of the questionnaire was limited, exploratory factor analysis provided some evidence that the questionnaire is a useful tool for measuring patient understanding of trial results. The developed questionnaire can be tailored for use in other trials in future examinations of patients understanding of trial results. This would provide insight into patient understanding and provide further validation data.

Thirdly, all SWATparticipants were aged 65 and over. The layout, format and language of this patient-based approach which was identified and developed may only be relevant for this study population. Other trial populations may prefer to receive the results via email, online or in person from a member of the study team ^12^. The evidence on patient preferences of receiving trial results is limited, therefore further research is needed to explore patient preferences of receiving trial results amongst different study populations.

It is also important to point out that the control group in this study received a copy of the trial results in a press release format. Most trial participants do not receive this. While this control method was a step further than normal procedure, the researchers in this study felt this was appropriate. The information presented in the press release was similar to that of the patient-based approach. However, the format and layout of the press release was different. Information was writtern in four long paragraphs separated by individual headings. It was also much shorter (1 page in total) that the patient-based approach (3 pages in total). Given the fact that press releases are written by public relations professionals with a view to communicating effectively and efficiently, this may have potentially minimised differences between the intervention and control conditions. The primary outcome of this study was assessing the impact of PPI on patient understanding of results, however, this was not the only potential impact. In hindsight, we adopted a limited approach to PPI in this study as we did not involve our PPI partners from the outset of the SWAT. Involving PPI partners in the development of core outcome sets for this SWAT could have identified other more appropriate primary outcome measures ^41^.

The aim of this SWAT was to investigate methods of disseminating trial findings to trial participants by using a PPI approach to identify, develop and evaluate a patient-based method of receiving trial results. The PPI approach actively involved focus group participants in making decisions about the result method and worked with PPI partners to co-develop the result letter. However, PPI partners were not involved in other aspects of the research process such as research design, data collection or analysis. This is partly due to the fact that PPI is a relatively new concept in clinical trials. As the majority of the literature has only been published in the last 12 months, there is little evidence available on the impact of PPI and no gold standard or comprehensive guidelines for researchers to follow ^29^. Thornton ^2^ suggests that in order for PPI to develop it is important to record its social and cultural history by collecting comprehensive databases and undertaking ongoing reviews of the impact of PPI. This paper along with the study protocol have been written in adherence with the Guidelines for Reporting Involvement of Patients and the Public ^23^, thus providing templates for involving patients and the public in clinical trial design and development. This study is an important step forwards in documenting the process of conducting PPI as part of a SWAT and evaluating its impact. Future research is needed to further develop PPI in clinical trial settings. As there is currently no gold standard or comprehensive guidelines for researchers to follow when evaluating the impact of PPI, further research is needed. This research should involve PPI partners in the development of core outcome sets for evaluating PPI impact. These would significantly enhance the literature in the area.

## Conclusion

Patient and Public Involvement (PPI) is advocated for every step of the trial process. We have demonstrated that it is feasible to involve PPI partners in the development of dissemination materials. Sharing clinical trial results with participants in a format understandable to laypersons will soon be a legal requirement ^11^. However, there is a significant lack of evidence as to the most appropriate methods of sharing results with participants. The study identified and developed a patient-based approach to disseminating clinical trial results for trial participants. Although, in this study PPI did not influence patients’ final understanding of results, it documents the process of conducting PPI within the clinical trial setting. This process may be useful for other trialists interested in conducting and evaluating the impact of PPI in clinical trials.

## Ethics approval and consent to participate

The research was approved in Ireland by the Clinical Research Ethics Committee of the Cork Teaching Hospitals, UCC, Ref ECM 4 (t).

All participants provided signed informed consent to take part in the study.

## Data availability

The raw data from this study cannot be sufficiently de-identified, and therefore are not publicly available. However, the data from the current study are available for further (collaborative) research purposes on reasonable request. Available datasets include transcripts from focus groups, field notes from PPI sessions and responses from the patient understanding questionnaire. To access the data, please contact the corresponding author ( emmy.racine@ucc.ie) or the Principal Investigator ( patricia.kearney@ucc.ie). Researchers must provide a written proposal on how the data will be used in research before access is granted.

## Supplementary material

All supplementary files are contained in one PDF document.

Supplementary File 1: Focus group topic guide

Supplementary File 2: Draft One of Patient-Based Result Letter

Supplementary File 3: Final Draft of Patient-Based Result Letter

Supplementary File 4: Standard Results Letter

Supplementary File 5: Patient Understanding Questionnaire

Supplementary File 6: Costs of Conducting PPI

## References

[1] INVOLVE: What is public involvement in research?2017 Reference Source

[2] Thornton H: Patient and public involvement in clinical trials. *BMJ.* 2008;336(7650):903–4. 10.1136/bmj.39547.586100.80 18436920 PMC2335270

[3] Rayment J Lanlehin R McCourt C: Involving seldom-heard groups in a PPI process to inform the design of a proposed trial on the use of probiotics to prevent preterm birth: a case study. *Res Involv Engagem.* 2017;3(1):11. 10.1186/s40900-017-0061-3 29062536 PMC5611657

[4] Shippee ND Domecq Garces JP Prutsky Lopez GJ: Patient and service user engagement in research: a systematic review and synthesized framework. *Health Expect.* 2015;18(5):1151–66. 10.1111/hex.12090 23731468 PMC5060820

[5] Buck D Gamble C Dudley L: From plans to actions in patient and public involvement: qualitative study of documented plans and the accounts of researchers and patients sampled from a cohort of clinical trials. *BMJ Open.* 2014;4(12):e006400. 10.1136/bmjopen-2014-006400 25475243 PMC4256646

[6] Dyer S: Rationalising public participation in the health service: the case of research ethics committees. *Health place.* 2004;10(4):339–48. 10.1016/j.healthplace.2004.08.004 15491894

[7] INVOLVE: Exploring the impact of public involvement on the quality of research: examples. Eastleigh;2013 Reference Source

[8] Dudley L Gamble C Allam A: A little more conversation please? Qualitative study of researchers' and patients' interview accounts of training for patient and public involvement in clinical trials. *Trials.* 2015;16(1):190. 10.1186/s13063-015-0667-4 25928689 PMC4410574

[9] Staniszewska S Herron-Marx S Mockford C: Measuring the impact of patient and public involvement: the need for an evidence base. *Int J Qual Health Care.*Oxford University Press;2008;20(6):373–4. 10.1093/intqhc/mzn044 18836184

[10] Long CR Stewart MK Cunningham TV: Health research participants’ preferences for receiving research results. *Clin Trials.* 2016;13(6):582–91. 10.1177/1740774516665598 27562368 PMC5286914

[11] Regulation (EU) No 536/2014 of the European Parliament and of the Council of 16 April 2014 on clinical trials on medicinal products for human use, and repealing Directive 2001/20/EC.2014 Reference Source

[12] Fernandez CV Kodish E Weijer C: Informing study participants of research results: an ethical imperative. *IRB.* 2003;25(3):12–9. 10.2307/3564300 14569989

[13] SPIRIT: The SPIRIT Statement.2018 Reference Source

[14] CONSORT: The CONSORT Statement.2018 Reference Source

[15] AllTrials: Find out more.2018 Reference Source

[16] Stott DJ Rodondi N Kearney PM: Thyroid Hormone Therapy for Older Adults with Subclinical Hypothyroidism. *N Engl J Med.* 2017;376(26):2534–2544. 10.1056/NEJMoa1603825 28402245

[17] O'Cathain A Murphy E Nicholl J: Why, and how, mixed methods research is undertaken in health services research in England: a mixed methods study. *BMC Health Serv Res.* 2007;7(1):85. 10.1186/1472-6963-7-85 17570838 PMC1906856

[18] Racine E Hurley C Cheung A: Study within a trial (SWAT) protocol. Participants' perspectives and preferences on clinical trial result dissemination: The TRUST Thyroid Trial experience. *Contemp Clin Trials Commun.* 2017;7:163–5. 10.1016/j.conctc.2017.07.001 29696180 PMC5898556

[19] Hartnett T: Consensus-oriented Decision-making: the CODM Model for Facilitating Groups to Widespread Agreement.Gabriola Island. New Society Publishers;2011 Reference Source

[20] Braun V Clarke V: Using thematic analysis in psychology. *Qual Res Psychol.* 2006;3(2):77–101. 10.1191/1478088706qp063oa

[21] Agency NAL: NALA's Plain English Editing and Training service.2017 Reference Source

[22] European Commission: Summary of Clinical Trial Results for Laypersons.Recommendations of the expert group on clinical trials for the implementation of Regulation (EU) No 536/2014 on clinical trials on medicinal products for human use. Consultation Document. European Commission;2016 Reference Source

[23] Staniszewska S Brett J Simera I: GRIPP2 reporting checklists: tools to improve reporting of patient and public involvement in research. *Res Involv Engagem.* 2017;3(1):13. 10.1186/s40900-017-0062-2 29062538 PMC5611595

[24] Cameron R Dwyer T Richardson S: Lessons from the field: Applying the good reporting of a mixed methods study (GRAMMS) framework. *Electronic Journal of Business Research Methods.* 2013;11(2):53–64. Reference Source

[25] Brett J Staniszewska S Mockford C: A systematic review of the impact of patient and public involvement on service users, researchers and communities. *Patient.* 2014;7(4):387–95. 10.1007/s40271-014-0065-0 25034612

[26] Brett J Staniszewska S Mockford C: The PIRICOM Study: A systematic review of the conceptualisation, measurement, impact and outcomes of patients and public involvement in health and social care research.2010 Reference Source

[27] Shalowitz DI Miller FG: Communicating the results of clinical research to participants: attitudes, practices, and future directions. *PLoS Med.* 2008;5(5):e91. 10.1371/journal.pmed.0050091 18479180 PMC2375946

[28] Kerasidou A: Sharing the Knowledge: Sharing Aggregate Genomic Findings with Research Participants in Developing Countries. *Dev World Bioeth.* 2015;15(3) :267–74. 10.1111/dewb.12071 25292263 PMC4632193

[29] Dalal H Wingham J Pritchard C: Communicating the results of research: how do participants of a cardiac rehabilitation RCT prefer to be informed? *Health Expect.* 2010;13(3):323–30. 10.1111/j.1369-7625.2009.00580.x 19906214 PMC5060540

[30] Snowdon C Garcia J Elbourne D: Reactions of participants to the results of a randomised controlled trial: exploratory study. *BMJ.* 1998;317(7150):21–6. 10.1136/bmj.317.7150.21 9651262 PMC28597

[31] Dixon-Woods M Jackson C Windridge KC: Receiving a summary of the results of a trial: qualitative study of participants' views. *BMJ.* 2006;332(7535):206–10. 10.1136/bmj.38675.677963.3A 16401631 PMC1352050

[32] Partridge AH Wong JS Knudsen K: Offering participants results of a clinical trial: sharing results of a negative study. *Lancet.* 2005;365(9463):963–4. 10.1016/S0140-6736(05)71085-0 15766998

[33] Domecq JP Prutsky G Elraiyah T: Patient engagement in research: a systematic review. *BMC Health Serv Res.* 2014;14(1):89. 10.1186/1472-6963-14-89 24568690 PMC3938901

[34] Mockford C Murray M Seers K: A SHARED study-the benefits and costs of setting up a health research study involving lay co-researchers and how we overcame the challenges. *Res Involv Engagem.* 2016;2(1):8. 10.1186/s40900-016-0021-3 29062509 PMC5611649

[35] Jinks C Carter P Rhodes C: Patient and public involvement in primary care research - an example of ensuring its sustainability. *Res Involv Engagem.* 2016;2(1):1. 10.1186/s40900-016-0015-1 29062502 PMC5611572

[36] Perkins R Goddard K: Reality out of the rhetoric: increasing user involvement in a mental health trust. *Ment Health Rev J.* 2004;9(1):21–4. 10.1108/13619322200400006

[37] INVOLVE: Payment for Involvement. 2010. Reference Source

[38] Brandberg Y Johansson H Bergenmar M: Patients' knowledge and perceived understanding–Associations with consenting to participate in cancer clinical trials. *Contemp Clin Trials Commun.* 2016;2:6–11. 10.1016/j.conctc.2015.12.001 29736441 PMC5935834

[39] Wilson P Mathie E Keenan J: ReseArch with Patient and Public invOlvement: a RealisT evaluation – the RAPPORT study.2015. 26378332

[40] Patrick JH Pruchno RA Rose MS: Recruiting research participants: a comparison of the costs and effectiveness of five recruitment strategies. *Gerontologist.* 1998;38(3):295–302. 10.1093/geront/38.3.295 9640849

[41] Williamson PR Altman DG Bagley H: The COMET Handbook: version 1.0. *Trials.* 2017;18(Suppl 3):280. 10.1186/s13063-017-1978-4 28681707 PMC5499094

