# Supplementary material for: Participants’ perspectives and preferences on clinical trial result dissemination: The TRUST Thyroid Trial experience
Source: HRB Open Res. 2019 Mar 22;1:14. Originally published 2018 Apr 11. [Version 2] doi: 10.12688/hrbopenres.12817.2 (PMC6973522; doi:10.12688/hrbopenres.12817.2)
Supplement: Supplementary file 1 [file hrbopenres-1-13965-s0000.tgz › 9993b55d-f634-45c0-87e3-288dcabd90e4.pdf]

Supplementary Material

Supplementary File 1: Focus group topic guide

Topic Guide

The objective of this study is to use a public and patient (PPI) strategy to develop a preferred method of receiving end-of-trial information for participants who were enrolled in the TRUST study. The aim is then to compare the information developed through the standard end-of-trail documents developed by the co-ordinating study site in Glasgow.

- Thank participants, introduce researcher & study.
- Briefly go through information sheet and consent form.
- Outline general housekeeping rules which will be said to participants at the beginning of the focus groups.

  -If it is ok with you, I will audio record this group discussion so I can give you my full attention. However, the research assistant will be taking field notes during the discussion just to ensure that we don't miss anything.

  -Everything we discuss will be confidential and your identity will remain anonymous. We may use direct quotes from this discussion but your identity and position will be kept completely anonymous and your name will not be used on any reports or publications.

  -You can choose to withdraw from the discussion at any time.

  -Do you have any questions before we get started?
- Sign consent and give copy.

Questions	Prompt/probe
1. Can you each give your first name only please?	-Distribute name tags
**Trial Experience**<br>2. How did you first become aware of the TRUST study?	Who told you about it? GP? Other healthcare provider? Advertising campaign? Other?
3. When you first heard about the trial what did you think?	Were you interested in the trial straight away?<br>Positive/Negative first impression?<br>Did you think it would be useful for you?
4. How was your experience of the TRUST Thyroid trial?	Tell me about your initial contact with your GP?<br>What was it like when you were first recruited?<br>How did you find the study visits? (research team, doctors etc.) Were they helpful?<br>How do you feel now that the trial has ended?

	What was your most positive and negative experience of the TRUST study?<br><br>How do you think this could be improved upon?
5. In your opinion do you think the information you received at study visits was informative?	Too much information? Too little? What other information would you have liked? Did you seek information from other sources-GP, internet, other? If so what kind of information?
6. During the trial, did you think you were on the placebo or the active drug?	If you had a choice at the beginning of the trial, which one would you have picked? Why?
7. Most participants have requested to be un-blinded. Why do you think this is?	Do you think this is important to know?<br>How would you feel if no one ever told you?
**Result Dissemination**<br>8. Do you want to find out the final study results? Why?	Do you think this is important? Why?
9. When the final results of the trial are known, how would you like to find out?	By post/telephone contact/email/face to face meeting?
10. In your opinion do you think study participants should be involved in formulating information leaflets for research studies?	Yes- in what capacity?<br><br>No- why not?
11. Would you be interested in helping to write the information leaflet that we will send to all participants about the final study results?	Is there anything in particular you would like to be included in this leaflet?
12. Would you participate in another research study?	Yes, why?<br>No, why?<br>Would you have any interest in being part of advisory groups for trials in the future?

Supplementary File 2: Draft One Patient-Preferred Result Letter

Dear Participant,

Thank you for taking part in the TRUST Thyroid Trial. You may be interested to read the results of the trial which are listed below in question and answer format.

1. **What is subclinical hypothyroidism (SCH)?**

Subclinical hypothyroidism also called mildly underactive thyroid. It affects around one in six people over the age of 65 and has been linked to various health problems, such as heart attacks and strokes, in later life. At this point in time doctors are not sure how to treat these patients because extensive research has never been done.

2. **How is SCH diagnosed?**

A person is said to have SCH if two of their blood tests, taken within a 3month period, show that their TSH level is persistently high (≥4.6 to ≤ 19.9Mu/L) and their free thyroxin (fT4) remains in normal range.

3. **What was the aim of the TRUST Thyroid Trial?**

To test if older community dwelling adults aged ≥65 years with subclinical hypothyroidism (SCH) benefit from Levothyroxine treatment. The main benefit the trial was looking at was an improvement in participant's Thyroid Specific Quality of Life. This was measured using the ThyPRO questionnaire.

4. **What were the secondary benefits examined the in trial?**

During your trial visits, you completed a number of questionnaires, physical tests and weight measurements. The purpose of these tests was to determine if Levothyroxine can prevent cardiovascular disease, improve health-related quality of life, muscle function and cognition in older adults with SCH.

5. **Who took part in the TRUST Thyroid Trial?**

In total 738 participants with SCH from Ireland, the United Kingdom, Netherlands and Switzerland took part in the study.

6. **Why was I chosen to take part in the trial?**

You were asked to take part in the trial as during a routine review of your blood result, your GP found you had an abnormal TSH result and so you would be suitable for the TRUST Thyroid Trial.

7. **How many participants were on the active drug-levothyroxine and how many participants were on the placebo?**

From the total 738 participants recruited to the trial X were on the active drug and X were on the placebo.

8. **How long was the TRUST Thyroid Trial?**

The trial ran from May 2013 to November 2016. Participants had to be in the trial for a minimum of 12months and a maximum of 36 months.

9. Why did the trial stop?

The TRUST trial stopped as it has reached its scheduled completion date.

10. What was the primary result of the TRUST Thyroid Trial?

The trial showed that participants on the active drug had (better or worse) Thyroid specific Quality of Life scores compared to the placebo group

11. What were the secondary results of the trial?

In total the trial included 8 secondary outcomes. The results of these tests are listed in the table below:

Outcome	Questionnaire	Results
	General QOL	
Handgrip strength	Jadaar hand dynamometer	
Cognitive function	Letter Digit Coding Test (LDCT)	
Total mortality and cardiovascular mortality		
Functional ability	Bathel Index and the Older American resources and services (OARS)	
Haemoglobin	Blood test at baseline and 1 year visit	
Blood pressure	Measured at screening and final visit	
Weight and waist circumference	Measured at screening and final visit	

12. Will these results change how treatment for SCH patients?

13. What should I do going forward

If you require more information please contact the TRUST team on (--) ------.

Supplementary File 3: Final Draft of Patient-Preferred Result Letter

Dear Participant,

Your participation in the TRUST Thyroid Trial has helped researchers answer important health questions which will be of benefit to many people in the future.

We are now sending you information about the trial and the final results of the trial. As you know, during the trial you attended a number of study visits. During these visits you completed a number of questionnaires, physical tests and weight measurements. We collected and analysed all of this information and now the final results of the trial are available.

We hope you will take the time to learn the results of the trial and we would like to sincerely thank you for taking part.

Kind regards,

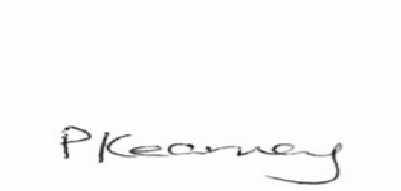

____

Professor Patricia M. Kearney
Principal Investigator TRUST Thyroid Trial
Dept. of Epidemiology and Public Health
University College Cork.

About the Trial…

What was the TRUST Thyroid Trial?

The Thyroid Hormone Replacement for Subclinical Hypothyroidism Trial (TRUST) was set up to better understand how to treat people with subclinical hypothyroidism.

Who was in charge of the trial?

The main study site for the trial was at the University of Glasgow, Scotland. The trial was funded by a €6 million grant from the EU's FP7 programme. This programme is the EU's main funding for research and development in Europe.

What was the aim of the TRUST Trial?

The purpose of the trial was to look at whether or not adults aged over 65 years old with subclinical hypothyroidism benefit from taking the active drug, levothyroxine. Levothyroxine replaces or provides extra thyroid hormone. Thyroid hormone is normally produced by the thyroid gland.

How long was the TRUST trial?

The trial started in May 2013 and ended in November 2016.

Who took part in the TRUST trial?

A total of 738 people took part in the trial from the following countries:

Ireland (115 people)

UK (150)

Netherlands (255)

Switzerland (218)

The hub centre for the Irish site was located at the Mercy University Hospital, Cork. There were also five other Irish sites located at Waterford University Hospital, Bantry General Hospital, Kerry General Hospital, St John's Hospital Limerick and Vista Primary Care Centre, Naas.

Why did you ask me to take part?

You were asked to take part because you are aged over 65 and a routine blood test at your doctor's surgery showed that you may have subclinical hypothyroidism (SCH).

About the Condition…

What is subclinical hypothyroidism (SCH)?

Subclinical hypothyroidism (SCH) is a mildly underactive thyroid. This means that your thyroid gland in your neck may not be producing the right amount of thyroid hormones.

What are the symptoms of SCH?

The condition often shows no symptoms or mild symptoms like:

- fatigue,
- depression
- memory problems
- cold intolerance
- consistent weight gain

In later life, the condition has also been linked to various health problems such as heart attacks and strokes.

How is SCH diagnosed?

There are two important hormones that the body needs for the thyroid to function properly. These hormones are called 'Thyroid Stimulating Hormone' (TSH) and Thyroxin (T4). Subclinical hypothyroidism (SCH) is diagnosed when a person's blood results show that their T4 levels are normal but their TSH level is mildly high (from 4.6 to 19.9 Mu/L).

How is SCH treated?

Before the TRUST trial, doctors were not sure how to treat SCH because previous research was not able to provide any answers.

About the Drug…

What is Levothyroxine?

Levothyroxine is used to treat hypothyroidism. It replaces or provides more thyroid hormone which is normally produced by the thyroid gland. This means that your body has enough thyroid hormone to maintain normal mental and physical activity.

What are the side effects of Levothyroxine?

Many people using this medication do not have any side effects. Most of the side effects are associated with hyperthyroidism (when the thyroid gland makes too much thyroxine). These include:

-temporary hair loss
-sweating
-difficulty sleeping
-vomiting and diarrhoea
-headaches
-weight loss
-chest pain
-high temperature
-flushing
-restlessness
-irregular/fast heartbeat
-muscle cramps

Please contact your GP if you have any questions about the side effects of Levothyroxine.

The Results of the TRUST Thyroid Trial.

How was the trial carried out?

After we asked you to take part in the study, we gave half of the participants the active drug (levothyroxine) and the other half a placebo. A placebo is a substance which has no active ingredient and therefore has no effect. During study visits, you will remember that you completed a number of questionnaires, physical tests and weight measurements. We collected and analysed the information and now are pleased to present you with the final overall results of the study**.** Please note that these are not your own personal results but the results of the study as a whole.

What were the results of the TRUST trial?

The results of the TRUST Thyroid Trial show that levothyroxine provides no apparent benefits for older people with subclinical hypothyroidism.

Further information

**Outcome**	**Results**
Thyroid specific quality of life	No differences found between placebo group and levothyroxine group.
Handgrip strength	No differences found between placebo group and levothyroxine group.
Cognitive function (ability to process thoughts and related to memory)	No differences found between placebo group and levothyroxine group.
Total mortality and cardiovascular mortality	No differences found between placebo group and levothyroxine group.
Functional ability (activities of daily living)	No differences found between placebo group and levothyroxine group.
Haemoglobin (part of red blood cells that carries oxygen)	No differences found between placebo group and levothyroxine group.
Blood pressure	No differences found between placebo group and levothyroxine group.
Weight and waist circumference (width)	No differences found between placebo group and levothyroxine group.

Should doctors treat people with subclinical hypothyroidism?

The results of the TRUST trial show that people with subclinical hypothyroidism do not benefit from taking levothyroxine. However, doctors should prescribe medication on a case-by-case basis.

What should I do now?

If you have any questions about your medical condition and whether or not you should be taking levothyroxine, please speak with your GP.

You can find more information about this study and its results:

Website: www.trustthyroidtrial.com **TRUST Telephone No:** (021) 4205595

Academic publication: http://www.nejm.org/doi/full/10.1056/NEJMoa1603825

Supplementary File 4: Standard Results Letter

TRIAL RESULTS

A mildly underactive thyroid gland (subclinical hypothyroidism) is a common condition in older age, affecting up to one-in-ten older men and women. According to current guidelines, nine of every ten women with the condition receive thyroid hormone tablets, typically levothyroxine, which has become the most prescribed drug in the USA and the third most prescribed drug in the UK. A large 5-year European study now shows that the common treatment of this condition with levothyroxine provides no apparent benefits, calling for a re-evaluation of the guidelines. The main results of the study were launched today with a publication in *The New England Journal of Medicine* along with simultaneous presentation at the Endocrine Society meeting (ENDO 2017) in Orlando, USA.

European 5-year study of 737 older adults
A team of researchers from four European Universities have followed 737 older adults (average age 74 years) to determine if levothyroxine provides clinical benefits for older people with subclinical hypothyroidism. This condition has been linked to various health problems in later life, such as tiredness or lethargy, problems with the blood circulation, muscle weakness, slowed speed of thinking, and increasing blood pressure and weight, but it is also argued that the condition causes little harm. Half of the older adults in the trial were allocated to a placebo and half to levothyroxine, and participants were followed up for at least a year. The 5-year study found that treatment with levothyroxine tablets did effectively restore a normal balance of thyroid function, but did not give any symptomatic benefits. There was also no improvement of muscle strength, speed of thinking or any effect on body weight or blood pressure. Specific advices for the oldest old (over 80 years old) will be available next year, when TRUST results will be combined with an ongoing trial among over 80s.

No worthwhile benefits from levothyroxine treatment
Based on these findings, the team concluded that there is now convincing evidence that older people with a mildly underactive thyroid do not get worthwhile benefits from levothyroxine treatment. Professor David Stott from the University of Glasgow, who led the international study, explains: “Our aim is to significantly improve the health and well-being of older people with subclinical hypothyroidism, by resolving uncertainties about how best to manage this condition. Treatment with levothyroxine is common in clinical practice, but controversial. Our study concludes this treatment provides no apparent benefits for older adults and should therefore no longer be started routinely for this condition. An update of the guidelines is necessary.”

About the TRUST research project
Thyroid Hormone Replacement for Subclinical Hypo-Thyroidism Trial (TRUST) is a European research project of experts in ageing, thyroid problems and vascular disease, investigating current treatment practices for people who suffer from a mildly underactive thyroid gland. Professor David Stott from Scotland leads the study, along with collaborators from the Netherlands (lead Professor Jacobijn Gussekloo), Switzerland (Professor Nicolas Rodondi), Ireland (Professor Patricia Kearney) and Denmark (Professor Rudi Westendorp). The data handling was performed at the Robertson Centre for Biostatistics at the University of Glasgow (lead Professor Ian Ford). The study was funded by the European Union and medicines were provided free of charge by Merck KGaA. Please view the **methods paper** and **protocol**.

The article ‘Thyroid Hormone Therapy for Older Adults with Subclinical Hypothyroidism’ by David Stott, Jacobijn Gussekloo, Nicolas Rodondi, Patricia Kearney, Rudi Westendorp et al. was published by The New England Journal of Medicine on 3 April 2017: ***http://www.nejm.org/doi/full/10.1056/NEJMoa1603825***

Supplementary File 5: Patient Understanding Questionnaire

TRUST

THYROID TRIAL

Questionnaire

Your Understanding of the TRUST Thyroid Trial.

Please read carefully:

We would like to know if the information we gave you about the TRUST Thyroid Trial was useful to you. This questionnaire asks some questions about the TRUST Thyroid Trial, levothyroxine and the results of the trial.

Subject's Understanding

- I understand that my participation is voluntary.
- I understand that I will not be identified by name in the final report.
- I am aware that all documents will be kept confidential in the secure possession of the researcher.
- I understand that I may withdraw from the study at any with no adverse repercussions.

Under the **Data Protection Acts 1998 and 2003** you are entitled to make an access request for a copy of your personal information relating to this study. If you wish to make a request for access to your data, please contact Professor Patricia Kearney (021) 4205502 or patricia.kearney@ucc.ie

If you agree to the above, please sign here:

Full Name: ____

Signature: __________________________________ **Date signed:** ________________

Plain English
Approved by NALA

For Office Use only:

Your understanding of the TRUST Thyroid Trial

The TRUST Trial looked at the effects of using the medicine levothyroxine to treat people with subclinical hypothyroidism. Subclinical hypothyroidism (SCH) is a mildly underactive thyroid. This means that the thyroid gland in the neck may not be producing the right amount of thyroid hormones. Please answer the following questions relating to the TRUST trial, levothyroxine and subclinical hypothyroidism.

Q.1 Please tick how much you agree with each statement

	Strongly Disagree	Disagree	Neither Agree or Disagree	Agree	Strongly Agree
I understand why the TRUST Thyroid Trial took place	◯	◯	◯	◯	◯
I understand why I was invited to take part in the TRUST Thyroid Trial	◯	◯	◯	◯	◯
I know why the medicine Levothyroxine is used to treat subclinical hypothyroidism	◯	◯	◯	◯	◯
I am aware of the side effects of Levothyroxine	◯	◯	◯	◯	◯
I understand the impact of Levothyroxine on thyroid-specific quality of life	◯	◯	◯	◯	◯
I understand how doctors will use the results of the TRUST Thyroid Trial to treat people with subclinical hypothyroidism	◯	◯	◯	◯	◯

Please tick <u>one</u> correct answer for each question

Q.2 The primary aim of the TRUST Thyroid Trial was to measure the impact of Levothyroxine on:

◯ Heart and circulatory system problems ◯ Thyroid-specific quality of life ◯ Metabolic rate and weight gain

Q.3 A common side effect linked to Levothyroxine is:

◯ Vision impairment ◯ Irregular heartbeat ◯ Increased sensitivity to the cold

Q.4 The results of the TRUST Thyroid Trial showed that Levothyroxine:

- ◯ Improves thyroid-specific quality of life
- ◯ Has no effect on thyroid-specific quality of life
- ◯ Disimproves thyroid-specific quality of life

Q.5 In the future, would you take Levothyroxine to treat your subclinical hypothyroidism?

◯ Yes ◯ No ◯ Need more information

Please explain your answer in the comment box below:

Q.6 Do you think doctors should prescribe Levothyroxine based on the results of the trial? Please read the following situation and tick yes or no.

Situation A

Mary is 65 years old. She went to the practise nurse to get a blood test to check her cholesterol. She told the nurse that she has been feeling tired lately and the nurse suggested checking her thyroid level as well. When the blood results returned, the doctor told her that her T4 level (the main thyroid hormone) was normal and her TSH level (a different thyroid hormone) was higher than normal at 8.5 Mu/L meaning she has subclinical hypothryoidism. Should the doctor prescribe Levothyroxine for Mary?

☐ Yes ☐ No

Situation B

John is 80 years old and has been quite forgetful lately. His daughter was worried about him and brought him to the doctor for a routine check-up. When his blood results returned, the doctor rang and told him that his T4 level was normal but his TSH level was high at 7.8. This meant that his thyroid gland was slightly underactive. Should they ask the doctor to prescribe Levothryoxine for him?

☐ Yes ☐ No

Please give reason(s) for your answer:

Thank you for taking the time to fill out this questionnaire.
Your views are important to us.

Supplementary File 6: Costs of Conducting PPI

Phase	PPI Activity	Description	Cost
ALL	Researcher Salary	Research Assistant (3 months)	6588.75
1	Focus Groups (3 separate sessions)	Catering costs	90
		Gift vouchers for participants	400
		Printing and Stationary	11.30
		Postage	27.36
1	Public and Patient Expert Sessions (4 sessions)	Refreshments for study participants	16.00
1	NALA review	Plain English Editing- PPI results letter	230
		Plain English Review –PPI results letter and questionnaire	197
3	Result Dissemination	Printing	17.50
		Postage	104
3	Questionnaire	Printing	36.05
		Postage	332
		**TOTAL COST**	**€8049.96**
