# Supplementary material for: Participants’ perspectives and preferences on clinical trial result dissemination: The TRUST Thyroid Trial experience
Source: HRB Open Res. 2019 Mar 22;1:14. Originally published 2018 Apr 11. [Version 2] doi: 10.12688/hrbopenres.12817.2 (PMC6973522; doi:10.12688/hrbopenres.12817.2)
Supplement: Peer review history

# Peer review: Reviewer response for version 2

**DOI:** 10.21956/hrbopenres.13965.r26546

**Published:** 2019-03-22

**Authors:** Shaun P. Treweek, Hanne Bruhn

**Affiliations:** 1 Health Services Research Unit (HSRU), Institute of Applied Health Sciences, School of Medicine, Medical Sciences and Nutrition, University of Aberdeen, Aberdeen, UK; 2 University of Aberdeen, Aberdeen, UK

We are happy with how the authors have addressed our suggestions and we have no further comments.

We confirm that we have read this submission and believe that we have an appropriate level of expertise to confirm that it is of an acceptable scientific standard.

# Peer review: Reviewer response for version 1

**DOI:** 10.21956/hrbopenres.13878.r26210

**Published:** 2018-05-16

**Authors:** Thilo Kroll, Marina Zaki

**Affiliations:** 1 UCD School of Nursing, Midwifery and Health Systems, University College Dublin, Dublin, Ireland

The paper is an important contribution to enhance patient and public involvement in trial research. A very worthy SWAT, one of a few SWATs conducted within the TRUST trial. Please explain SWAT at the outset.

**Some suggestions:**

 While the authors refer to the PPI definition by INVOLVE it would have been helpful to get a clearer understanding of the authors’ specific understanding and conceptualisation of PPI in the context of their study. The approach of involving patients in shaping the dissemination of results is laudable but admittedly quite limiting and appears to be an afterthought rather than an integral element of the trial. The paper very much resides in the traditional experimental (albeit using a preference-based design), researcher-led paradigm (clearly expressed in the ‘randomisation’ to determine effectiveness of modes of dissemination – which somewhat contravenes the notion of engaging with a diverse public and patient population for bespoke dissemination) with limited shift of control over the research process to patients or the public. For example, how were study participants involved at the intersection of analysis, dissemination and knowledge transfer (selection of outcomes that are relevant? Decision on subgroup analysis? Involvement in interpretation of analysis findings? Decisions about outlets for dissemination (audiences, events, settings, format and content?).

 It would be helpful to see a more detailed description of the PPI Group. How was this group selected, what is its composition, how frequently did it meet and what was its immediate influence in the research process throughout. Did members of the PPI group participate as co-researchers at any stage of contribute to the facilitation, analysis etc of the focus group results?

 Paper mentions qualitative aspect and discusses the need to ensure accurate and relevant information is being shared with participants. Wonder if benefit could be derived from drawing upon the notion of “translational statistics”, where additional statistical information is obtained, analysed and accurately translated to clinically meaningful findings - perhaps with the authors referring to the tension of statistical significance versus clinical relevance and the importance of minimising statistical jargon when presenting trial results. Great to see contribution of NALA – but did the authors experience this barrier of translating statistical results into findings that were clinically relevant, most notably in the context of PPI in understanding this? The suggestion therefore is that including PPI here helps to minimise both statistical and clinical jargon, which can often be challenging, for a concise and accurate report.

 Also, some of the language used appears to be a departure from the PPI approach. For example (in Section Phase 3), “This document highlights the information that trial participants **should understand** after reading their trial summary.” The intention of the authors is clear but the wording very much suggests a prescriptive approach on the part of the academic team.

 It would be important to distinguish between ‘literacy’ and ‘health literacy’. They represent related but substantially different concepts.

 In line with other members of the trial community, the paper could benefit from the word ‘transparency’ – paragraph 2 introduction – to put it in context with other papers discussing sharing of trial findings and results. The authors could also consider making reference to the AllTrials initiative in paragraph 2 (not directly relevant to PPI, but important nonetheless in the movement towards transparency in trials).

 It would be helpful to clarify how the CODM was specifically used in the process and to provide evidence for the step by step approach.

 Methods section of Phase 2 - “ One member of the research team was un-blinded in order to perform the randomisation and distribute the results of the trial.” – what other parts of the study was this member involved in? Did their unblinding have any impact on any other aspects of the study?

 The paper could benefit from briefly mentioning the ‘moral obligation’ trialists have to share results – in this sense, I mean results to be the trial findings – (results in the context of data sharing is subject to new EU GDPR, which is something the authors could consider to mention but unlikely to have a place for discussion in this article; justification for not being able to share the data is already briefly mentioned in the “data availability” paragraph). So the term ‘moral obligation’ of trialists would complement the qualitative quotes the authors have in the results section about patients feeling they contributed to research.

 Phase 1 results part: “This feeling of contributing for a collective benefit was further reinforced when participants discussed their desire to understand how the results of the trial will be implemented by medical experts and ultimately how it will affect others who have the condition:” à could mention one aspect, particularly this being a drug trial, of how statistics can explain why one treatment works on some patients and not others etc and that this may be something public and patients would like to know, therefore is important to accurately portray.

 “education” – demographic not discussed in detail in other sections of paper- only mentioned in terms of not being statistically significantly different – need more? Relevance to PPI is not explained thoroughly and should be strengthened

 Great mention of CT regulation and checklist when reporting PPI – but could also benefit from mentioning the fundamental reporting guidance in trials, such as putting SPIRIT into context and relevance, if any, to layman’s terms and similar to using CONSORT as a reference and explaining this to PPI? Also refer to www.clinicaltrials.gov reporting section for public

 Clearly, it is a strength of the SWAT having participants already from the trial - see the trial through from beginning to end so their help is important in terms of accurate context/background when interpreting and disseminating findings

 Generally, well-written, well laid out, concise and important paper.

We confirm that we have read this submission and believe that we have an appropriate level of expertise to confirm that it is of an acceptable scientific standard.

# Response

**Published:** 2019-01-11

**Authors:** Emmy Racine

**Affiliations:** University College Cork, Ireland

**The paper is an important contribution to enhance patient and public involvement in trial research. A very worthy SWAT, one of a few SWATs conducted within the TRUST trial. Please explain SWAT at the outset.**

Firstly, thank you for your comprehensive comments. 

To address this, we have inserted the following sentence into the abstract: 

“The aim of this Study Within A Trial (SWAT) is to use….” (Abstract)

**While the authors refer to the PPI definition by INVOLVE it would have been helpful to get a clearer understanding of the authors’ specific understanding and conceptualisation of PPI in the context of their study. The approach of involving patients in shaping the dissemination of results is laudable but admittedly quite limiting and appears to be an afterthought rather than an integral element of the trial. The paper very much resides in the traditional experimental (albeit using a preference-based design), researcher-led paradigm (clearly expressed in the ‘randomisation’ to determine effectiveness of modes of dissemination – which somewhat contravenes the notion of engaging with a diverse public and patient population for bespoke dissemination) with limited shift of control over the research process to patients or the public. For example, how were study participants involved at the intersection of analysis, dissemination and knowledge transfer (selection of outcomes that are relevant? Decision on subgroup analysis? Involvement in interpretation of analysis findings? Decisions about outlets for dissemination (audiences, events, settings, format and content?).**

We have reflected on our approach to PPI in this Study Within a Trial and have added the following text to the manuscript to address this comment:

The aim of this SWAT was to investigate methods of disseminating trial findings to trial participants by using a PPI approach to identify, develop and evaluate a patient-based method of receiving trial results. The PPI approach actively involved focus group participants in making decisions about the result method and working with PPI partners to co-develop the result letter. However, PPI partners were not involved in other aspects of the research process such as research design, data collection or analysis. This is partly due to the fact that PPI is a relatively new concept in clinical trials. As the majority of the literature has only been published in the last 12 months, there is little evidence available on the impact of PPI and no gold standard or comprehensive guidelines for researchers to follow (30). (Discussion: Strengths and Limitations, Paragraph 5)

**It would be helpful to see a more detailed description of the PPI Group. How was this group selected, what is its composition, how frequently did it meet and what was its immediate influence in the research process throughout. Did members of the PPI group participate as co-researchers at any stage of contribute to the facilitation, analysis etc of the focus group results?**

A more detailed description of the PPI group has been added to the manuscript to address this comment:

A PPI group was established to develop and refine the content of the patient-based approach for the dissemination of results. During the focus groups, three TRUST trial participants volunteered to take part in the PPI group. In addition to these three PPI partners, an additional partner was identified from a previous qualitative research study undertaken by the research team. This individual was keen to learn more about research and expressed an interested in being involved in future projects. While this individual had previous experience of taking part in research (as an interview participant), she had no experience of taking part in a clinical trial or being involved as a PPI partner.(Methods: PPI group)

There were four. PPI partners in total (three trial participants and one older adult).  Each partner took part in one one-to-one session (Results: PPI group)

Members of the PPI group did not participate as co-researchers in the facilitation, analysis etc. of focus group results.  We have addressed our approach to PPI in the previous comment and have made necessary additions to the main manuscript. 

**Paper mentions qualitative aspect and discusses the need to ensure accurate and relevant information is being shared with participants. Wonder if benefit could be derived from drawing upon the notion of “translational statistics”, where additional statistical information is obtained, analysed and accurately translated to clinically meaningful findings - perhaps with the authors referring to the tension of statistical significance versus clinical relevance and the importance of minimising statistical jargon when presenting trial results. Great to see contribution of NALA – but did the authors experience this barrier of translating statistical results into findings that were clinically relevant, most notably in the context of PPI in understanding this? The suggestion therefore is that including PPI here helps to minimise both statistical and clinical jargon, which can often be challenging, for a concise and accurate report.**

While we believe this is an important point, we believe it was not a major contributor in our study. As there were no statistically significant or clinically relevant results, the results of the trial were simple and straightforward (ie. no effect), and so we did not have this challenge.

**Also, some of the language used appears to be a departure from the PPI approach. For example (in Section Phase 3), “This document highlights the information that trial participants should understand after reading their trial summary.” The intention of the authors is clear but the wording very much suggests a prescriptive approach on the part of the academic team.**

The authors do not mean to sound prescriptive in their approach. This sentence has been changed to the following:

‘This document highlights the information which should be presented to trial participants in the trial summary at the end of a trial’. (Methods: Evaluation)

**It would be important to distinguish between ‘literacy’ and ‘health literacy’. They represent related but substantially different concepts.**

Both concepts were important in this study. We collaborated with the National Adult Literacy Agency to ensure that the material in the results letter to trial participants was sufficiently easy to read and understand (literacy). This would help to ensure that trial participants were able to make sound health decisions based on the information presented to them (health literacy). 

To ensure accuracy, we have changed the wording throughout the document to ‘adult literacy’ and described our approach as follows: 

Adult literacy review

While the PPI group had significant input into the format and language used in the patient-based approach. The research team felt that it would be of additional benefit to collaborate with the National Adult Literacy Agency (NALA) to ensure the document adhered to national “Plain English” standards. These standards ensured that the information presented to trial participants was sufficiently easy to read and understand (literacy). This would help to ensure that trial participants were able to make sound health decisions based on the information presented (health literacy) (15). This review was an iterative process with several drafts exchanged for editing. (L-P-) 

**In line with other members of the trial community, the paper could benefit from the word ‘transparency’ – paragraph 2 introduction – to put it     in context with other papers discussing sharing of trial findings and results. The authors could also consider making reference to the AllTrials initiative in paragraph 2 (not directly relevant to PPI, but important nonetheless in the movement towards transparency in trials).**

Yes we agree, this paragraph would benefit from a reference to the recent movement towards transparency in trials. The following text has been added: 

The increasing understanding of the importance of sharing research results with study participants is somewhat linked to a wider movement towards transparency in trials. This movement is largely promoted by initiatives such as SPIRIT, CONSORT and AllTrials. The SPIRIT Statement provides guidance to researchers to improve the completeness and quality of trial protocols (11), the  Consolidated Standards of Reporting Trials (CONSORT) Statement is an evidence based, minimum set of recommendations for reporting randomised trials (12) and the  AllTrials initiative calls for all past and present trials to be registered and their full methods and summary results reported (13). Some of these initiatives also include recommendations for disseminating results to research participants. For example, the SPIRIT statement states that study results must be released to participating physicians, referring physicians, patients and the general medical community (11). (Introduction, Paragraph 2).

**It would be helpful to clarify how the CODM was specifically used in the process and to provide evidence for the step by step approach.**

The following text has been added to the manuscript to describe how the CODM model was specifically used in this study:

The Consensus-Oriented-Decision-Making (CODM) model was used to guide the group to reach a consensus. The CODM model is accepted as a flexible model for reaching decisions  (14). In this study some of the steps were initiated by the focus group facilitator and others occurred naturally as a follow on from the previous step. Below is an outline of each of the seven steps of the CODM model and how they were used in this study:

1.    Framing the topic: The focus group facilitator introduced the idea of sharing results with participants and provided some context on the reasons why results are/ are not shared with participants.

2.    Open discussion: The facilitator asked the group whether or not they think results should be shared with trial participants and whether or not they would like to receive the results of the TRUST trial. 

3.    Identifying underlying concerns: The previous discussion naturally followed on to participants asking questions and expressing concerns about the result method, content and language that would be used. 

4.    Collaborative proposal building: The group worked together to agree on the important elements of the results in terms of result method, content and language.

5.    Choosing a direction: This step occurred naturally as part of the previous step. 

6.    Synthesizing a final proposal: The facilitator re-iterated the proposal the group had agreed upon and asked the group for feedback.

7.    Closure: This step occurred naturally as part of the previous step. (Methods: Phase 1)

**Methods section of Phase 2 - “ One member of the research team was un-blinded in order to perform the randomisation and distribute the results of the trial.” – what other parts of the study was this member involved in? Did their unblinding have any impact on any other aspects of the study?**

This member of the research team (AC) was involved in the design of the study, preparing study materials, performing data collection and developing the result material. After they were un-blinded in order to perform the randomisation and distribute the results, they were not involved in the data analysis or interpretation in any way. The following sentence has been added to the manuscript to provide clarity on this:

“As they were un-blinded to perform these two important tasks, they were not involved in the data analysis or interpretation in any way.” (Methods: Phase 2)

**The paper could benefit from briefly mentioning the ‘moral obligation’ trialists have to share results – in this sense, I mean results to be the trial findings – (results in the context of data sharing is subject to new EU GDPR, which is something the authors could consider to mention but unlikely to have a place for discussion in this article; justification for not being able to share the data is already briefly mentioned in the “data availability” paragraph). So the term ‘moral obligation’ of trialists would complement the qualitative quotes the authors have in the results section about patients feeling they contributed to research.**

We have inserted the following text into the discussion to address this:

“Focus group findings showed that participants felt that receiving results would provide an acknowledgement of their individual contribution to the trial. This finding complements previous commentaries about result sharing being an ‘ethical imperative or ‘moral obligation’. Fernandez et al. points out that many participants place their trust in science and researchers owe a debt to participants to fulfil their trust and recognise their altruism (10, 23).” (Discussion, Paragraph 2)

We have not mentioned the EU GDPR in this paper as we feel it is not directly relevant to sharing trial results with participants. The upcoming EU Clinical Trial regulation is mentioned in the introduction which will be the main EU regulation which requires investigators to share clinical trial results with participants in a format understandable to laypersons. 

**Phase 1 results part: “This feeling of contributing for a collective benefit was further reinforced when participants discussed their desire to understand how the results of the trial will be implemented by medical experts and ultimately how it will affect others who have the condition:” à could mention one aspect, particularly this being a drug trial, of how statistics can explain why one treatment works on some patients and not others etc and that this may be something public and patients would like to know, therefore is important to accurately portray.**

We acknowledge that understanding how statistics can explain why one treatment works on some patients and not others is an important aspect and may be something that patients would like to know. However, this aspect was not brought up by the trial participants in this study (either during the focus groups or PPI sessions). As this paper was about listening to trial participants and giving them the information they wanted to know, we feel it would be inappropriate to add it in to the paper at this late stage and presume that it is something that they wanted to know.  Therefore, while it is an important aspect, we have not made any changes to address it in our paper.

**“education” – demographic not discussed in detail in other sections of paper- only mentioned in terms of not being statistically significantly different – need more? Relevance to PPI is not explained thoroughly and should be strengthened**

We have added the following text to the discussion to address this: 

Previous research suggests that people that actively choose to engage in research either as research participants or involvement partners are more likely to be middle-class and highly educated (34, 35). In this study, those that attended the focus groups and PPI group were similar in education level to those that did not attend. This is not surprising considering the entire study sample had already actively volunteered to take part in the TRUST trial.  (Discussion: Strengths and Limitations, Paragraph 1)

**Great mention of CT regulation and checklist when reporting PPI – but could also benefit from mentioning the fundamental reporting guidance in trials, such as putting SPIRIT into context and relevance, if any, to layman’s terms and similar to using CONSORT as a reference and explaining this to PPI? Also refer to reporting section for public**

The following text has been inserted into the introduction to address this comment:

The increasing understanding of the importance of sharing research results with study participants is somewhat linked to a wider movement towards transparency in trials. This movement is largely promoted by initiatives such as SPIRIT, CONSORT and AllTrials. The SPIRIT Statement provides guidance to researchers to improve the completeness and quality of trial protocols (11), the  Consolidated Standards of Reporting Trials (CONSORT) Statement is an evidence based, minimum set of recommendations for reporting randomised trials (12) and the  AllTrials initiative calls for all past and present trials to be registered and their full methods and summary results reported (13). Some of these initiatives also include recommendations for disseminating results to research participants. For example, the SPIRIT statement states that study results must be released to participating physicians, referring physicians, patients and the general medical community (11). (Introduction, Paragraph 2)

**Clearly, it is a strength of the SWAT having participants already from the trial - see the trial through from beginning to end so their help is important in terms of accurate context/background when interpreting and disseminating findings**

We have made the following changes to the manuscript to address this:

This section of the discussion has been reworded from ‘Limitations of the study’ to ‘Strengths and limitations of the study’ (Discussion)

This paragraph in question has been rephrased as a highlight of the SWAT and moved to the first paragraph in the ‘Strengths and limitations of the study’ section. It now reads as following:

“While this study has provided important insights into patients’ preferences of receiving trial results, it is not without limitations. Firstly, existing PPI literature states that ‘to understand the research needs and challenges, PPI has to engage people who are able to offer perspectives from the study population’ (3). All PPI partners in this study were active members of the research community as they had taken part in the TRUST trial and had agreed for long-term follow up. This is a major strength of the SWAT as they were able to offer perspectives from the study population, however it does have an important implication for their reporting of understanding the results of the trial. They may be more inclined to rate their understanding as high because of their investment in the trial (31), thus potentially minimising differences between the intervention and control conditions and minimising inferences that can be drawn about the intervention.”  Discussion: Strengths and Limitations, Paragraph 1)

**Generally, well-written, well laid out, concise and important paper.**

# Response

**Published:** 2019-01-11

**Authors:** Emmy Racine

**Affiliations:** University College Cork, Ireland

**The paper is an important contribution to enhance patient and public involvement in trial research. A very worthy SWAT, one of a few SWATs conducted within the TRUST trial. Please explain SWAT at the outset.**

Firstly, thank you for your comprehensive comments.

To address this, we have inserted the following sentence into the abstract:

“The aim of this Study Within A Trial (SWAT) is to use….” (Abstract)

**While the authors refer to the PPI definition by INVOLVE it would have been helpful to get a clearer understanding of the authors’ specific understanding and conceptualisation of PPI in the context of their study. The approach of involving patients in shaping the dissemination of results is laudable but admittedly quite limiting and appears to be an afterthought rather than an integral element of the trial. The paper very much resides in the traditional experimental (albeit using a preference-based design), researcher-led paradigm (clearly expressed in the ‘randomisation’ to determine effectiveness of modes of dissemination – which somewhat contravenes the notion of engaging with a diverse public and patient population for bespoke dissemination) with limited shift of control over the research process to patients or the public. For example, how were study participants involved at the intersection of analysis, dissemination and knowledge transfer (selection of outcomes that are relevant? Decision on subgroup analysis? Involvement in interpretation of analysis findings? Decisions about outlets for dissemination (audiences, events, settings, format and content?).**

We agree that our approach to PPI in this study was limited. We involved trial participants in shaping the dissemination of results and did not involve them in other stages of the research process. We agree that there was a limited shift of control over the research process to PPI partners. We have reflected on our limited approach to PPI and have added the following text to the manuscript to address this comment:

The aim of this SWAT was to investigate methods of disseminating trial findings to trial participants by using a PPI approach to identify, develop and evaluate a patient-based method of receiving trial results. The PPI approach actively involved focus group participants in making decisions about the result method and working with PPI partners to co-develop the result letter. However, PPI partners were not involved in other aspects of the research process such as research design, data collection or analysis. This is partly due to the fact that PPI is a relatively new concept in clinical trials. As the majority of the literature has only been published in the last 12 months, there is little evidence available on the impact of PPI and no gold standard or comprehensive guidelines for researchers to follow (30). (Discussion: Strengths and Limitations, Paragraph 5)

This study is an important step forwards in documenting the process of conducting PPI as part of a SWAT and evaluating its impact. (Discussion: Strengths and Limitations, Paragraph 5)

**It would be helpful to see a more detailed description of the PPI Group. How was this group selected, what is its composition, how frequently did it meet and what was its immediate influence in the research process throughout. Did members of the PPI group participate as co-researchers at any stage of contribute to the facilitation, analysis etc of the focus group results?**

A more detailed description of the PPI group has been added to the manuscript to address this comment:

A PPI group was established to develop and refine the content of the patient-based approach for the dissemination of results. During the focus groups, three TRUST trial participants volunteered to take part in the PPI group. In addition to these three PPI partners, an additional partner was identified from a previous qualitative research study undertaken by the research team. This individual was keen to learn more about research and expressed an interested in being involved in future projects. While this individual had previous experience of taking part in research (as an interview participant), she had no experience of taking part in a clinical trial or being involved as a PPI partner. (Methods: PPI group).

There were four. PPI partners in total (three trial participants and one older adult). Each partner took part in one one-to-one session (Results: PPI group).

Members of the PPI group did not participate as co-researchers in the facilitation, analysis etc. of focus group results.  We have addressed our limited approach to PPI in the previous comment and have made necessary additions to the main manuscript.

**Paper mentions qualitative aspect and discusses the need to ensure accurate and relevant information is being shared with participants. Wonder if benefit could be derived from drawing upon the notion of “translational statistics”, where additional statistical information is obtained, analysed and accurately translated to clinically meaningful findings - perhaps with the authors referring to the tension of statistical significance versus clinical relevance and the importance of minimising statistical jargon when presenting trial results. Great to see contribution of NALA – but did the authors experience this barrier of translating statistical results into findings that were clinically relevant, most notably in the context of PPI in understanding this? The suggestion therefore is that including PPI here helps to minimise both statistical and clinical jargon, which can often be challenging, for a concise and accurate report.**

While we believe this is an important point, we believe it was not a major contributor in our study. As there were no statistically significant or clinically relevant results, the results of the trial were simple and straightforward (ie. no effect), and so we did not have this challenge.

**Also, some of the language used appears to be a departure from the PPI approach. For example (in Section Phase 3), “This document highlights the information that trial participants should understand after reading their trial summary.” The intention of the authors is clear but the wording very much suggests a prescriptive approach on the part of the academic team.**

The authors do not mean to sound prescriptive in their approach. This sentence has been changed to the following:

‘This document highlights the information which should be presented to trial participants in the trial summary at the end of a trial’. (Methods: Evaluation)

**It would be important to distinguish between ‘literacy’ and ‘health literacy’. They represent related but substantially different concepts.**

Both concepts were important in this study. We collaborated with the National Adult Literacy Agency to ensure that the material in the results letter to trial participants was sufficiently easy to read and understand (literacy). This would help to ensure that trial participants were able to make sound health decisions based on the information presented to them (health literacy).

To ensure accuracy, we have changed the wording throughout the document to ‘adult literacy’ and described our approach as follows:

**Adult literacy review**

While the PPI group had significant input into the format and language used in the patient-based approach. The research team felt that it would be of additional benefit to collaborate with the National Adult Literacy Agency (NALA) to ensure the document adhered to national “Plain English” standards. These standards ensured that the information presented to trial participants was sufficiently easy to read and understand (literacy). This would help to ensure that trial participants were able to make sound health decisions based on the information presented (health literacy) (15). This review was an iterative process with several drafts exchanged for editing.

**In line with other members of the trial community, the paper could benefit from the word ‘transparency’ – paragraph 2 introduction – to put it           in context with other papers discussing sharing of trial findings and results. The authors could also consider making reference to the AllTrials initiative in paragraph 2 (not directly relevant to PPI, but important nonetheless in the movement towards transparency in trials).**

Yes we agree, this paragraph would benefit from a reference to the recent movement towards transparency in trials. The following text has been added:

The increasing understanding of the importance of sharing research results with study participants is somewhat linked to a wider movement towards transparency in trials. This movement is largely promoted by initiatives such as SPIRIT, CONSORT and AllTrials. The SPIRIT Statement provides guidance to researchers to improve the completeness and quality of trial protocols (11), the  Consolidated Standards of Reporting Trials (CONSORT) Statement is an evidence based, minimum set of recommendations for reporting randomised trials (12) and the  AllTrials initiative calls for all past and present trials to be registered and their full methods and summary results reported (13). Some of these initiatives also include recommendations for disseminating results to research participants. For example, the SPIRIT statement states that study results must be released to participating physicians, referring physicians, patients and the general medical community (11). (Introduction, Paragraph 2).

**It would be helpful to clarify how the CODM was specifically used in the process and to provide evidence for the step by step approach.**

The following text has been added to the manuscript to describe how the CODM model was specifically used in this study:

The Consensus-Oriented-Decision-Making (CODM) model was used to guide the group to reach a consensus. The CODM model is accepted as a flexible model for reaching decisions  (14). In this study some of the steps were initiated by the focus group facilitator and others occurred naturally as a follow on from the previous step. Below is an outline of each of the seven steps of the CODM model and how they were used in this study:

1. Framing the topic: The focus group facilitator introduced the idea of sharing results with participants and provided some context on the reasons why results are/ are not shared with participants.
2. Open discussion: The facilitator asked the group whether or not they think results should be shared with trial participants and whether or not they would like to receive the results of the TRUST trial.
3. Identifying underlying concerns: The previous discussion naturally followed on to participants asking questions and expressing concerns about the result method, content and language that would be used.
4. Collaborative proposal building: The group worked together to agree on the important elements of the results in terms of result method, content and language.
5. Choosing a direction: This step occurred naturally as part of the previous step.
6. Synthesizing a final proposal: The facilitator re-iterated the proposal the group had agreed upon and asked the group for feedback.
7. Closure: This step occurred naturally as part of the previous step. (Methods: Phase 1)

 

**Methods section of Phase 2 - “ One member of the research team was un-blinded in order to perform the randomisation and distribute the results of the trial.” – what other parts of the study was this member involved in? Did their unblinding have any impact on any other aspects of the study?**

This member of the research team (AC) was involved in the design of the study, preparing study materials, performing data collection and developing the result material. After they were un-blinded in order to perform the randomisation and distribute the results, they were not involved in the data analysis or interpretation in any way. The following sentence has been added to the manuscript to provide clarity on this:

“As they were un-blinded to perform these two important tasks, they were not involved in the data analysis or interpretation in any way.” (Methods: Phase 2)

**The paper could benefit from briefly mentioning the ‘moral obligation’ trialists have to share results – in this sense, I mean results to be the trial findings – (results in the context of data sharing is subject to new EU GDPR, which is something the authors could consider to mention but unlikely to have a place for discussion in this article; justification for not being able to share the data is already briefly mentioned in the “data availability” paragraph). So the term ‘moral obligation’ of trialists would complement the qualitative quotes the authors have in the results section about patients feeling they contributed to research.**

We have inserted the following text into the discussion to address this:

“Focus group findings showed that participants felt that receiving results would provide an acknowledgement of their individual contribution to the trial. This finding complements previous commentaries about result sharing being an ‘ethical imperative or ‘moral obligation’. Fernandez et al. points out that many participants place their trust in science and researchers owe a debt to participants to fulfil their trust and recognise their altruism (10, 23).” (Discussion, Paragraph 2)

We have not mentioned the EU GDPR in this paper as we feel it is not directly relevant to sharing trial results with participants. The upcoming EU Clinical Trial regulation is mentioned in the introduction which will be the main EU regulation which requires investigators to share clinical trial results with participants in a format understandable to laypersons.

**Phase 1 results part: “This feeling of contributing for a collective benefit was further reinforced when participants discussed their desire to understand how the results of the trial will be implemented by medical experts and ultimately how it will affect others who have the condition:” à could mention one aspect, particularly this being a drug trial, of how statistics can explain why one treatment works on some patients and not others etc and that this may be something public and patients would like to know, therefore is important to accurately portray.**

We acknowledge that understanding how statistics can explain why one treatment works on some patients and not others is an important aspect and may be something that patients would like to know. However, this aspect was not brought up by the trial participants in this study (either during the focus groups or PPI sessions). As this paper was about listening to trial participants and giving them the information they wanted to know, we feel it would be inappropriate to add it in to the paper at this late stage and presume that it is something that they wanted to know.  Therefore, while it is an important aspect, we have not made any changes to address it in our paper.

**“education” – demographic not discussed in detail in other sections of paper- only mentioned in terms of not being statistically significantly different – need more? Relevance to PPI is not explained thoroughly and should be strengthened**

We have added the following text to the discussion to address this:

Previous research suggests that people that actively choose to engage in research either as research participants or involvement partners are more likely to be middle-class and highly educated (34, 35). In this study, those that attended the focus groups and PPI group were similar in education level to those that did not attend. This is not surprising considering the entire study sample had already actively volunteered to take part in the TRUST trial.  (Discussion: Strengths and Limitations, Paragraph 1)

**Great mention of CT regulation and checklist when reporting PPI – but could also benefit from mentioning the fundamental reporting guidance in trials, such as putting SPIRIT into context and relevance, if any, to layman’s terms and similar to using CONSORT as a reference and explaining this to PPI? Also refer to reporting section for public**

The following text has been inserted into the introduction to address this comment:

The increasing understanding of the importance of sharing research results with study participants is somewhat linked to a wider movement towards transparency in trials. This movement is largely promoted by initiatives such as SPIRIT, CONSORT and AllTrials. The SPIRIT Statement provides guidance to researchers to improve the completeness and quality of trial protocols (11), the  Consolidated Standards of Reporting Trials (CONSORT) Statement is an evidence based, minimum set of recommendations for reporting randomised trials (12) and the  AllTrials initiative calls for all past and present trials to be registered and their full methods and summary results reported (13). Some of these initiatives also include recommendations for disseminating results to research participants. For example, the SPIRIT statement states that study results must be released to participating physicians, referring physicians, patients and the general medical community (11). (Introduction, Paragraph 2)

**Clearly, it is a strength of the SWAT having participants already from the trial - see the trial through from beginning to end so their help is important in terms of accurate context/background when interpreting and disseminating findings**

We have made the following changes to the manuscript to address this:

This section of the discussion has been reworded from ‘Limitations of the study’ to ‘Strengths and limitations of the study’ (Discussion)

This paragraph in question has been rephrased as a highlight of the SWAT and moved to the first paragraph in the ‘Strengths and limitations of the study’ section. It now reads as following:

“While this study has provided important insights into patients’ preferences of receiving trial results, it is not without limitations. Firstly, existing PPI literature states that ‘to understand the research needs and challenges, PPI has to engage people who are able to offer perspectives from the study population’ (3). All PPI partners in this study were active members of the research community as they had taken part in the TRUST trial and had agreed for long-term follow up. This is a major strength of the SWAT as they were able to offer perspectives from the study population, however it does have an important implication for their reporting of understanding the results of the trial. They may be more inclined to rate their understanding as high because of their investment in the trial (31), thus potentially minimising differences between the intervention and control conditions and minimising inferences that can be drawn about the intervention.”  Discussion: Strengths and Limitations, Paragraph 1)

**Generally, well-written, well laid out, concise and important paper.**

# Peer review: Reviewer response for version 1

**DOI:** 10.21956/hrbopenres.13878.r26194

**Published:** 2018-04-30

**Authors:** Shaun P. Treweek, Hanne Bruhn

**Affiliations:** 1 Health Services Research Unit (HSRU), Institute of Applied Health Sciences, School of Medicine, Medical Sciences and Nutrition, University of Aberdeen, Aberdeen, UK; 2 University of Aberdeen, Aberdeen, UK

This research article reports a Study Within A Trial (SWAT) hosted by the Thyroid Hormone Replacement for Subclinical Hypo-Thyroidism Trial (TRUST). The TRUST trial is an international trial and the SWAT was only conducted in one of the participating countries, namely Ireland. The purpose of the SWAT was to use a ‘Patient and Public Involvement (PPI) approach’ to identify and develop results reporting for the TRUST trial to its participants and subsequently evaluate the result materials. Evaluation of the result materials was carried out by assessing recipients’ understanding of the trial results compared to result materials as provided by the lead TRUST site. The SWAT protocol was published last year.

 Although the study is small and has some limitations it is an important piece in the result provision literature for trials, providing the detailed account of a thorough approach to the results provision process in trials.

 We have a few comments which should be addressed:

**Overall** 

 Some people wonder why it is necessary to quantify at all the contribution public and patients make to a trial given that they are clearly trial stakeholders and we don’t do this for other stakeholders such as the statistician and the trial manager. Other people definitely do think it is necessary to quantify the gain from PPI. It would be good if the authors could mention this somewhere in the article. It could be early as a rationale for doing the study, or the Discussion in recognition of the different views.   

 - Identifying the intervention results as ‘patient-preferred method’ is at times confusing (it is easily misunderstood as the mode of delivery). Alternatives could be ‘patient-based approach’, ‘patient-generated materials’ or ‘patient-centred approach’. 

**Abstract**

 Background – rather than referring to ‘an increasing consensus’ the  ‘increasingly recognised as an essential component’ text mentioned in the Introduction in the main text seems easier to agree with.  

 Results – it would be better rephrase so that it is clear the results are specific to the TRUST trial participants rather than in general.  For example ‘TRUST patients want to receive…’

 Results – it is stated that “TRUST participants (n=101) were randomised to the intervention.” Unless we have misunderstood something, this must be a mistake as this is the total available sample to be randomised so not all of them can get the intervention. Our guess is that 101 were involved in the SWAT evaluation and that 51 were randomised to the intervention.

 Conclusion – please rephrase “conduct PPI” to e.g. ‘We have demonstrated that it is feasible to involve PPI members in the development of result dissemination materials.’

 Conclusion – The reference to age in “The study identified and developed a patient-preferred method of receiving clinical trial results for older adults over 65 years.” wasn’t part of the aim of the study as far as we can see and the authors should reconsider including it in the conclusion. If developing a strategy for the over 65s was a distinct aim of the study, then the text of study aims needs tweaked.   

 Conclusion – it would be good if the authors could see if they can improve “,… it provides a record of the process of conducting PPI within the clinical trial setting.” We’re not entirely clear what is meant. 

**Main text**

 Methods – please ensure that the term used to denote the control/comparison group is consistent  throughout the paper as currently these terms are used interchangeably (also in tables and figures).

 In the results section it is stated that the PPI group consisted of three trial participants and one older adult. It would be good to give a sentence in Methods on how this ‘older adult’ was identified and recruited.

 The published protocol doesn’t say that the materials will also reviewed by the National Adult Literacy Agency (NALA). That this is an addition should be mentioned, along with why.  It’s not a problem, just that it would be good to be clear.

**Results**

 Table 1 - Clarify the table title and include that it is the ‘characteristics of the trial participants stratified by participation in the different stages of the study’.

 Table 1 - Some of the column headings need clarification.

 “TRUST participants (n=104)” it should be clear these are the Irish TRUST sample only. It should also be noted that three participants were not eligible for the rest of the study as they took part in the PPI group.

 “Attended focus groups (n=19), Total sample n=38 RR=50%” it should be clear only 38 were invited/eligible or that this is a sub-sample of the 104.

 Please add an explanation of what RR is short for.

 “Randomised into intervention (n=101)”, it should be clear n=101 were randomised (delete “into intervention”). See earlier comment about this.  It’s involved in SWAT evaluation that has n=101 as far as we can see, not randomised to intervention.

 Main text relating to figure 1 – please include the denominator in the parentheses as the percentages and n can be misleading on their own when small numbers are concerned. 

 Figure 1 – in the title to figure 1, please clarify that patient understanding is presented by group. Please also include a short indication of how understanding was assessed (e.g. Likert scale).

 Table 2 – in the title include that this is presented by group and please also indicate how this was assessed (e.g. Likert scale).

 Any amendments which cannot be accommodated within the journal 15 word limit for the title of tables and figures could be added to a legend.

 In relation to the cost – it should also be clear that the NALA review is included in the cost.

**Discussion**

 Paragraph 2 – please clarify the description of the intervention in relation to the literature as it currently confounds mode of delivery with only one type of material that can be posted to participants.

 Paragraph 3 – please change ‘negative reaction’ to ‘negative impact’ or similar – the reaction might be negative in researchers’ view but completely justified. You should also comment on the trial context in relation to this as the TRUST trial is relatively low risk compared to some of the trials cited.

 Paragraph 4 relates to the aim of the SWAT and should probably be moved to a bit earlier in the Discussion, paragraph 2 perhaps.

   

 Paragraph 5 and 6 (about cost) – The main message seems to be that PPI needs to be planned and costed appropriately – please be clear about this. We’re not sure this sentence is necessary “As the primary focus of this study was to carry out PPI, researchers carefully considered the costs at grant proposal stage and wrote these costs into the budget.”

 - The authors paid a fixed cost for travel costs for PPI participation rather than to cover participants’ time.  The UK’s INVOLVE has recommendations for covering PPI participants’ time and it might be nice to link these to the sentences that follow about the institutional difficulties of actually making these payments (which colleagues have experienced at our own institution).

 - Please include some consideration of the cost of carrying out the SWAT – is it a lot of money for a trial and is it money well spent? Please also consider that there may be some hidden costs e.g. expert input into questionnaire development an expert input in to the qualitative phase. This kind of expertise will not be available to all trials.

 Paragraph 7 (Limitations of the study) – The sample size of the SWAT was limited but to n=104 not n=115 (11 had withdrawn before the SWAT started so were never eligible).

 Paragraph 8 (Limitations of the study) – Change “Secondly, all trial participants were aged 65 and over.” To ‘Secondly, all SWAT participants were aged 65 and over.’ While the former is true the SWAT did not include all trial participants.

 - Change “Alternative study populations may prefer…” to ‘Other trial populations may prefer…’

 Paragraph 8 (Limitations of the study) – This paragraph actually highlights a strength of the SWAT which is that the PPI members consulted were participants in the trial. We’d suggest highlighting that many SWATs will be underpowered because they are generally unable to change the size of the host trial; they are made for meta-analysis really.

 Paragraph 10 – This is an important consideration. In relation to this it would be beneficial to include the research team’s reflection on what is the most appropriate primary outcome to measure regarding dissemination of trial results. The use of PPI clearly had an impact in that the TRUST trial would not have posted results to Irish participants at all if it had not been for this SWAT (i.e. the Press release control for the SWAT was more than planned for the trial without the SWAT). 

 - It would be informative to have the authors comment on the difference in content of the two versions of the results used in the SWAT – was there a notable difference?

 - Would the authors in hindsight consider asking their PPI members what they think the primary outcome in the evaluation of the results materials should have been?

 What would the authors recommend for future evaluations of PPI impact?

 Paragraph 11 – It would be good to expand on “While this study provides a record of the process of conducting PPI as part of a SWAT, future research is needed to further develop PPI in clinical trial settings.” Any thoughts on what sort of PPI research?

We confirm that we have read this submission and believe that we have an appropriate level of expertise to confirm that it is of an acceptable scientific standard.

# Response

**Published:** 2019-01-11

**Authors:** Emmy Racine

**Affiliations:** University College Cork, Ireland

**This research article reports a Study Within A Trial (SWAT) hosted by the Thyroid Hormone Replacement for Subclinical Hypo-Thyroidism Trial (TRUST). The TRUST trial is an international trial and the SWAT was only conducted in one of the participating countries, namely Ireland. The purpose of the SWAT was to use a ‘Patient and Public Involvement (PPI) approach’ to identify and develop results reporting for the TRUST trial to its participants and subsequently evaluate the result materials. Evaluation of the result materials was carried out by assessing recipients’ understanding of the trial results compared to result materials as provided by the lead TRUST site. The SWAT protocol was published last year.**

****

**Although the study is small and has some limitations it is an important piece in the result provision literature for trials, providing the detailed account of a thorough approach to the results provision process in trials.**

****

**We have a few comments which should be addressed:**

**Some people wonder why it is necessary to quantify at all the contribution public and patients make to a trial given that they are clearly trial stakeholders and we don’t do this for other stakeholders such as the statistician and the trial manager. Other people definitely do think it is necessary to quantify the gain from PPI. It would be good if the authors could mention this somewhere in the article. It could be early as a rationale for doing the study, or the Discussion in recognition of the different views.**

Firstly, thank you for your comprehensive comments.

We agree this is a potentially contentious area. The following text has been added to the introduction of the manuscript as a rationale for doing the study.

“Research funders increasingly expect that PPI is prioritised and resourced within studies. This increasing expectation has heightened the risk of researchers carrying out ‘tick-box’ PPI rather than ‘meaningful’ involvement (6). There are many moral and ethical arguments being made for PPI. Many believe that as citizens and taxpayers, members of the public have a right to influence research that is being funded by public money (7). PPI researchers are also making pragmatic arguments for PPI and providing anecdotal accounts about how PPI can make research more relevant, accessible and acceptable to participants (8). The ethical arguments are often seen as sufficient regardless of any pragmatic impact. However, PPI costs time and money, therefore pragmatic claims need scrutiny (9). More substantive evidence is needed to evaluate the potential impact of PPI on the conduct and outcomes of research (5, 7)”. (Introduction, Paragraph 1)

**Identifying the intervention results as ‘patient-preferred method’ is at times confusing (it is easily misunderstood as the mode of delivery). Alternatives could be ‘patient-based approach’, ‘patient-generated materials’ or ‘patient-centred approach’.**

We have made the following changes throughout the manuscript:

“patient- preferred” has been changed to “patient-based”.

“patient-preferred method” has been changed to “patient-based approach”.

“Standard method” has been changed to “standard approach”.

**Abstract**

**Background – rather than referring to ‘an increasing consensus’ the ‘increasingly recognised as an essential component’ text mentioned in the Introduction in the main text seems easier to agree with.**

There may be a slight misunderstanding here. The ‘increasing consensus’ phrase in the abstract refers to the sharing of results with participants. The ‘increasingly recognised as an essential component’ phrase in the introduction refers to PPI in clinical research. These phrases are referring to two different things. We believe it would be confusing to the reader to use the same phrase to refer to two separate issues. Therefore, we have not made any changes to address this comment. We hope this is acceptable.

**Results – it would be better rephrase so that it is clear the results are specific to the TRUST trial participants rather than in general.  For example ‘TRUST patients want to receive…’**

This sentence in the abstract has been changed to:

“TRUST patients want to receive…” (Abstract)

**Results – it is stated that “TRUST participants (n=101) were randomised to the intervention.” Unless we have misunderstood something, this must be a mistake as this is the total available sample to be randomised so not all of them can get the intervention. Our guess is that 101 were involved in the SWAT evaluation and that 51 were randomised to the intervention.**

The sentence in the abstract has been corrected: 

“TRUST participants (n=101) were randomised to the either the intervention (n=51) or control group (n=50).” (Abstract)

**Conclusion – please rephrase “conduct PPI” to e.g. ‘We have demonstrated that it is feasible to involve PPI members in the development of result dissemination materials.’**

We have changed this sentence in the abstract as suggested:

“We have demonstrated that it is feasible to involve PPI partners in the development of result dissemination materials.” (Abstract)

We have also changed the same sentence in the main manuscript as following:

“We have demonstrated that it is feasible to involve PPI partners in the development of result dissemination materials.” (Conclusion, Paragraph 1)

**Conclusion – The reference to age in “The study identified and developed a patient-preferred method of receiving clinical trial results for older adults over 65 years.” wasn’t part of the aim of the study as far as we can see and the authors should reconsider including it in the conclusion. If developing a strategy for the over 65s was a distinct aim of the study, then the text of study aims needs tweaked.**

The aim of the study has been changed to clarify that all TRUST participants were aged 65 and over:

“”The aim of this Study Within A Trial (SWAT) is to use a patient and public involvement (PPI) approach to identify, develop and evaluate a patient-based approach to receiving trial results for participants in the Thyroid Hormone Replacement for Subclinical Hypo-Thyroidism Trial (TRUST), a trial of thyroxine versus placebo in people aged 65 years and older” (Abstract)

               

The conclusion has been changed to reflect the aim:

“The study identified, developed and evaluated a patient-based approach to receiving results for trial participants.” (Abstract)

**Conclusion – it would be good if the authors could see if they can improve “,… it provides a record of the process of conducting PPI within the clinical trial setting.” We’re not entirely clear what is meant.**

We have made changes to this sentence in both the abstract and the main manuscript to cliarfiy the conclusion: 

“… it documents the process of conducting PPI within the clinical trial setting. This process may be useful for other trialists interested in conducting and evaluating the impact of PPI in clinical trials.” (Abstract, Discussion, Paragraph 11 and Conclusion, Paragraph 1)

**Main text**

**Methods – please ensure that the term used to denote the control/comparison group is consistent  throughout the paper as currently these terms are used interchangeably (also in tables and figures).**

To ensure consistency, the word ‘comparison’ has been changed to ‘control’ throughout the manuscript.

**In the results section it is stated that the PPI group consisted of three trial participants and one older adult. It would be good to give a sentence in Methods on how this ‘older adult’ was identified and recruited.**

The following sentence has been added to the manuscript to give more detail on how the older adult was identified:

“In addition to these three PPI partners, an additional partner was identified from a previous qualitative research study undertaken by the research team. This older adult was keen to learn more about research and expressed an interested in being involved in future projects. While this individual had previous experience of taking part in research (as an interview participant), she had no experience of taking part in a clinical trial or being involved as a PPI partner”. (Methods: PPI group)

**The published protocol doesn’t say that the materials will also reviewed by the National Adult Literacy Agency (NALA). That this is an addition should be mentioned, along with why.  It’s not a problem, just that it would be good to be clear.**

We have inserted two additional sentences into the manuscript to clarify:

Sentence 1: While the PPI group had significant input into the format and language used in the patient-based approach, the research team felt that it would be of additional benefit to collaborate with the National Adult Literacy Agency (NALA) to ensure the document adhered to national “Plain English” standards. (Methods: Adult Literacy Review)

Sentence 2: Although the review was taken as an additional step to the published protocol for the study, the research team felt it was helpful to further ensure that the document was accessible and easy to understand. (Methods: Adult Literacy Review)

**Results**

**Table 1 - Clarify the table title and include that it is the ‘characteristics of the trial participants stratified by participation in the different stages of the study’.**

The table title has been clarified:

“Table 1: Characteristics of trial participants stratified by participation in the different stages of the study”. (Results: Table 1)

**Table 1 - Some of the column headings need clarification.**

**“TRUST participants (n=104)” it should be clear these are the Irish TRUST sample only. It should also be noted that three participants were not eligible for the rest of the study as they took part in the PPI group.**

The first column heading has been changed to:

“Total Irish TRUST participants (n=104)” (Results: Table 1)

A footnote has been added below the table to clarify this:

**^3^** Total Irish TRUST participants (n=104) excluding PPI partners (n=3)= n=101. (Results: Table 1)

**“Attended focus groups (n=19), Total sample n=38 RR=50%” it should be clear only 38 were invited/eligible or that this is a sub-sample of the 104.**

A footnote has been added to the table to clarify this:

**^1^**A subgroup of Irish TRUST participants (n=38) were invited to focus groups. (Results: Table 1)

**Please add an explanation of what RR is short for.**

A footnote has been added to the table to clarify this. `

^2^RR=Response Rate (Results: Table 1)

**“Randomised into intervention (n=101)”, it should be clear n=101 were randomised (delete “into intervention”). See earlier comment about this.  It’s involved in SWAT evaluation that has n=101 as far as we can see, not randomised to intervention.**

Yes, we agree that this needs to be clearer.  We have deleted ‘to intervention’ as you have suggested.

**Main text relating to figure 1 – please include the denominator in the parentheses as the percentages and n can be misleading on their own when small numbers are concerned.**

The main text relating to figure has now been changed to include the denominator in the parentheses.

**Figure 1 – in the title to figure 1, please clarify that patient understanding is presented by group. Please also include a short indication of how understanding was assessed (e.g. Likert scale).**

The title of Figure 1 has been changed to:

“Patient understanding of primary aim, side effect and trial result of the TRUST Thyroid Trial presented by group.” (Results: Figure 1)

A footnote has been added to the Figure 1 title to clarify how understanding was assessed. The footnote reads:

^“1 ^Patient understanding of primary aim, side effect and trial results was assessed using multiple choice questions.” (Results: Figure 1)

**Table 2 – in the title include that this is presented by group and please also indicate how this was assessed (e.g. Likert scale).**

The title for Table 2 has been changed in the manuscript to:

“Table 2: Patient perceptions of understanding presented by group.” (Results: Table 2)

A footnote has been added to the title to clarify how perceptions of understandings were assessed. The footnote reads:

“Patient perceptions of understanding were assessed using a five point LIKERT scale.” (Results: Table 2)

**Any amendments which cannot be accommodated within the journal 15 word limit for the title of tables and figures could be added to a legend.**

Thank you for this suggestion.

**In relation to the cost – it should also be clear that the NALA review is included in the cost.**

           

The following sentence has been added to make it clear that the NALA review is included in the cost: 

These costs included researcher salary, travel and expenses for PPI participants, adult literacy review and printing/postage costs. (Methods: Costs of conducting PPI)

**Discussion**

**Paragraph 2 – please clarify the description of the intervention in relation to the literature as it currently**

**confounds mode of delivery with only one type of material that can be posted to participants.**

****The following text has been added to this paragraph to clarify the description of the literature cited:

“A previous study investigating the preferences of individuals taking part in a cardiac rehabilitation trial found that 80% of trial participants (mean age 68.5 years) preferred to receive the results by post (22)” (Discussion, Paragraph 3)

**Paragraph 3- please change ‘negative reaction’ to ‘negative impact’-the reaction might be negative in researchers’ view but completely justified. You should also comment on the trial context in relation to this as the TRUST trial is relatively low risk compared to some other trials cited.**

We have changed the phrasing of ‘negative reaction’ to ‘negative impact’ and added the following sentence:

“Previous studies exploring participants’ reactions found that sharing trial results with participants can cause some negative impacts such as anxiety, anger, guilt, upset and confusion (23-25). As far as researchers in this study are aware, providing results did not cause any negative impacts. This may have been due to the fact that the TRUST trial had a low risk of morbidity or mortality compared to some of the other studies citing negative impacts.” (Discussion, Paragraph 4)

**Paragraph 4 relates to the aim of the SWAT and should probably be moved to a bit earlier in the Discussion, paragraph 2 perhaps.**

Paragraph 4 has been moved to the first paragraph of the discussion.

**Paragraph 5 and 6 (about cost) – The main message seems to be that PPI needs to be planned and costed appropriately – please be clear about this. We’re not sure this sentence is necessary “As the primary focus of this study was to carry out PPI, researchers carefully considered the costs at grant proposal stage and wrote these costs into the budget.”**

Yes this is the main message. We have deleted the sentence as suggested and added the following sentence to the paragraph:

“It is extremely important that researchers plan PPI at the grant proposal stage and estimate the costs appropriately.” (Discussion, Paragraph 5)

**The authors paid a fixed cost for travel costs for PPI participation rather than to cover participants’ time.  The UK’s INVOLVE has recommendations for covering PPI participants’ time and it might be nice to link these to the sentences that follow about the institutional difficulties of actually making these payments (which colleagues have experienced at our own institution).**

We have edited the text in this paragraph to include a reference to INVOLVE’s payment policy:

“INVOLVE, the national advisory group supporting active public involvement in health services, public health and social care research in the UK, have recommended that PPI partners should be paid for their involvement (30). Despite this, existing research suggests that institutional difficulties make negotiating the mechanisms of paying participants very difficult (32).” (Discussion, Paragraph 6)

**Please include some consideration of the cost of carrying out the SWAT – is it a lot of money for a trial and is it money well spent? Please also consider that there may be some hidden costs e.g. expert input into questionnaire development an expert input in to the qualitative phase. This kind of expertise will not be available to all trials.**

****

We have inserted the following text into the manuscript to address this:

“This breakdown provides a template to other researchers who plan to carry out and evaluate PPI as part of their research. It is important to note that not all costs associated with carrying out the study were included in this amount. For example, the only salary costed was that of the research assistant. The expertise provided by other members of the study team were not included in the total cost as they were being paid by the University or other research grants. The total cost of conducting this study was €8,049 which is not insignificant but should be considered in the context of the cost of large scale trials.” (Discussion, Paragraph 6)

**Paragraph 7 (Limitations of the study) – The sample size of the SWAT was limited but to n=104 not n=115 (11 had withdrawn before the SWAT started so were never eligible).**

We have changed the sample size to n=104. (Discussion: Strengths and Limitations, Paragraph 2)

**Paragraph 8 (Limitations of the study) – Change “Secondly, all trial participants were aged 65 and over.”**

**To ‘Secondly, all SWAT participants were aged 65 and over.’ While the former is true the SWAT did not include all trial participants.**

We have changed the manuscript as suggested. (Discussion: Strengths and Limitations, Paragraph 3)

**Change “Alternative study populations may prefer…” to ‘Other trial populations may prefer…’**

We have changed the manuscript as suggested.

**Paragraph 8 (Limitations of the study) – This paragraph actually highlights a strength of the SWAT which is that the PPI members consulted were participants in the trial. We’d suggest highlighting that many SWATs will be underpowered because they are generally unable to change the size of the host trial; they are made for meta-analysis really.**

This section of the discussion has been reworded from ‘Limitations of the study’ to ‘Strengths and limitations of the study’ (Discussion)

This paragraph in question has been rephrased as a highlight of the SWAT and moved to the first paragraph in the ‘Strengths and limitations of the study’ section. It now reads as following:

“While this study provides important insights into patients’ preferences of receiving trial results, it is not without limitations. Firstly, existing PPI literature states that ‘to understand the research needs and challenges, PPI has to engage people who are able to offer perspectives from the study population’ (3). All PPI partners in this study were active members of the research community as they had taken part in the TRUST trial and had agreed to long-term follow up. This is a strength of the SWAT as they were able to offer perspectives from the study population, however it does have an important implication for their reporting of understanding the results of the trial. They may be more inclined to rate their understanding as high because of their investment in the trial (31), thus potentially minimising differences between the intervention and control conditions and minimising inferences that can be drawn about the intervention.”

**Paragraph 10 – This is an important consideration. In relation to this it would be beneficial to include the research team’s reflection on what is the most appropriate primary outcome to measure regarding dissemination of trial results. The use of PPI clearly had an impact in that the TRUST trial would not have posted results to Irish participants at all if it had not been for this SWAT (i.e. the Press release control for the SWAT was more than planned for the trial without the SWAT).**

We agree that PPI had an important role to play in ensuring that participants actually received the results of the trial and other primary outcome measures could have been used to evaluate the impact of PPI. If we had involved PPI partners from the outset of this study, relevant outcome measures could have been co-developed by researchers and PPI partners. We have added this important point to the discussion as following:

The primary outcome of this study was assessing the impact of PPI on patient understanding of results, however, this was not the only potential impact. In hindsight, we adopted a limited approach to PPI in this study as we did not involve our PPI partners from the outset of the SWAT. Involving PPI partners in the design of the SWAT could have identified other appropriate primary outcome measures which would be more relevant to evaluating the impact of PPI in clinical trials (39). (Discussion: Strengths and Limitations, Paragraph 4)

**It would be informative to have the authors comment on the difference in content of the two versions of the results used in the SWAT – was there a notable difference?**

Both versions of the results have been uploaded as supplementary files and are accessible on the HRB Open platform.

“The intervention group (n=51) received the patient-based letter format (see Supplementary File 3: Final version patient-based results letter) and the control group (n=50) received a copy of the TRUST results press release, which was made available by the lead study site on the TRUST Thyroid Trial Website (see Supplementary File 4: Standard results letter).” (Results: Phase Two)

We have added a discussion on the difference in content of the versions of the results:

“It is also important to point out that the control group in this study received a copy of the trial results in a press release format. Most trial participants do not receive this.  While this control method was a step further than normal procedure, the researchers in this study felt this was appropriate. The information presented in the press release was similar to that of the patient-based approach. However, the format and layout of the press release was different. Information was written in four long paragraphs separated by individual headings.  It was also much shorter (1 page in total) than the patient-based approach (3 pages in total). Given the fact that press releases are written by public relations professionals with a view to communicating effectively and efficiently, this again may have potentially minimised differences between the intervention and control conditions.” (Discussion: Strengths and Limitations, Paragraph 4)

**Would the authors in hindsight consider asking their PPI members what they think the primary outcome in the evaluation of the results materials should have been?**

Yes. This is a very important suggestion. We have inserted the following text into the manuscript:

The primary outcome of this study was assessing the impact of PPI on patient understanding of results, however, this was not the only potential impact. In hindsight, we adopted a limited approach to PPI in this study as we did not involve our PPI partners from the outset of the SWAT. Involving PPI partners in the development of core outcome sets for this SWAT could have identified other more appropriate primary outcome measures (39). (Discussion: Strengths and Limitations, Paragraph 4)

**What would the authors recommend for future evaluations of PPI impact?**

We have considered our recommendations for future evaluations of PPI impact and have inserted the following text into the manuscript:

Evaluating the impact of PPI can be resource intensive, especially if PPI impact is not the primary aim of a study. As there is currently no gold standard or comprehensive guidelines for researchers to follow when evaluating the impact of PPI, further research is needed. This research should involve PPI partners in the development of core outcome sets for evaluating PPI impact. This would significantly enhance the literature in the area. (Discussion: Strengths and Limitations, Paragraph 5) 

**Paragraph 11 – It would be good to expand on “While this study provides a record of the process of conducting PPI as part of a SWAT, future research is needed to further develop PPI in clinical trial settings.” Any thoughts on what sort of PPI research?**

We have added our thoughts on the sort of PPI research needed in the discussion:

Evaluating the impact of PPI can be resource intensive, especially if PPI impact is not the primary aim of a study. As there is currently no gold standard or comprehensive guidelines for researchers to follow when evaluating the impact of PPI, further research is needed. This research should involve PPIpartners in the development of core outcome sets for evaluating PPI impact. This would significantly enhance the literature in the area. (Discussion: Strengths and Limitations, Paragraph 5)
